# F-actin-rich contractile endothelial pores prevent vascular leakage during leukocyte diapedesis through local RhoA signalling

**DOI:** 10.1038/ncomms10493

**Published:** 2016-01-27

**Authors:** Niels Heemskerk, Lilian Schimmel, Chantal Oort, Jos van Rijssel, Taofei Yin, Bin Ma, Jakobus van Unen, Bettina Pitter, Stephan Huveneers, Joachim Goedhart, Yi Wu, Eloi Montanez, Abigail Woodfin, Jaap D. van Buul

**Affiliations:** 1 Department of Molecular Cell Biology, Sanquin Research and Landsteiner Laboratory, Academic Medical Centre, University of Amsterdam, 1066CX Amsterdam, The Netherlands; 2 Genetics and Developmental Biology, Center for Cell Analyses and Modelling, University of Connecticut Health Centre, Farmington, Connecticut 06032, USA; 3 Experimental Medicine and Pharmacology, Centre for Microvascular Research, William Harvey Research Institute, Barts and The London School of Medicine and Dentistry, Queen Mary, University of London, Charterhouse Square, London, EC1M 6BQ, UK; 4 Molecular Cytology, Swammerdam Institute for Life Sciences, University of Amsterdam, Amsterdam 1098XH, The Netherlands; 5 Department of Angiogenesis, Walter-Brendel-Center of Experimental Medicine Ludwig-Maximilians University Marchioninistr. 27 81377 Munich, Germany

## Abstract

During immune surveillance and inflammation, leukocytes exit the vasculature through transient openings in the endothelium without causing plasma leakage. However, the exact mechanisms behind this intriguing phenomenon are still unknown. Here we report that maintenance of endothelial barrier integrity during leukocyte diapedesis requires local endothelial RhoA cycling. Endothelial RhoA depletion *in vitro* or Rho inhibition *in vivo* provokes neutrophil-induced vascular leakage that manifests during the physical movement of neutrophils through the endothelial layer. Local RhoA activation initiates the formation of contractile F-actin structures that surround emigrating neutrophils. These structures that surround neutrophil-induced endothelial pores prevent plasma leakage through actomyosin-based pore confinement. Mechanistically, we found that the initiation of RhoA activity involves ICAM-1 and the Rho GEFs Ect2 and LARG. In addition, regulation of actomyosin-based endothelial pore confinement involves ROCK2b, but not ROCK1. Thus, endothelial cells assemble RhoA-controlled contractile F-actin structures around endothelial pores that prevent vascular leakage during leukocyte extravasation.

## Abstract (web summary)

Endothelial cells can support leukocyte extravasation without causing vascular leakage, but the exact mechanism underlying this process has not been fully elucidated. Here the authors show that it is regulated through actomyosin-based endothelial pore confinement, which requires local endothelial RhoA activation.

## Graphical abstract

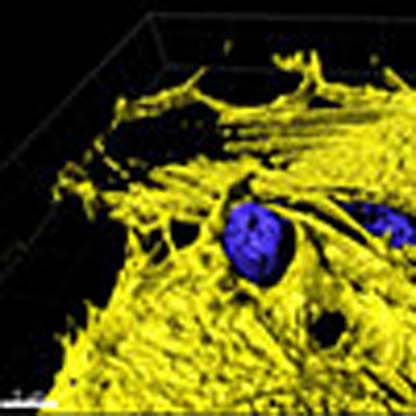

The clinical signs of inflammation, redness, heat, swelling and pain are caused by the acute inflammatory response including increased vasodilatation, enhanced microvascular permeability and leukocyte recruitment. During inflammation the endothelial barrier becomes more permissive for large molecules, leading to local plasma proteins leakage and oedema formation. Whether leukocyte transendothelial migration (TEM) directly causes increased microvascular permeability has been controversial for decades. Certain studies suggested leukocyte adhesion and transmigration to be the critical events leading to tissue damage and organ failure during inflammation and ischemia reperfusion^1^,^2^, since neutrophil depletion or CD11-/CD18-blocking antibodies have been shown to attenuate vascular injury under these circumstances^2^,^3^,^4^,^5^. However, when microvascular permeability was measured simultaneously with leukocyte–endothelial interactions, local plasma leakage sites were often different from those of leukocyte adhesion or transmigration^6^,^7^,^8^,^9^,^10^,^11^. Recently, it has been shown that intravenous injection of tumour necrosis factor (TNF)-α caused significant leukocyte adhesion and transmigration but did not affect basal microvessel permeability^12^. Moreover, several studies have shown that the timing of leukocyte adhesion and transmigration are not well linked with the evoked permeability change during acute inflammation^13^,^14^,^15^,^16^. Most of the abovementioned studies are descriptive, molecular evidence for the uncoupling between leukocyte TEM and vascular permeability has been recently shown by Wessel and colleagues. They mechanistically uncoupled leukocyte extravasation and vascular permeability by showing that opening of endothelial junctions in those distinct processes are controlled by different tyrosine residues of VE-cadherin *in vivo*^17^. However, how the endothelium maintains a tight barrier during leukocyte transendothelial migration is still unknown^18^.

Here we investigate the mechanism by which endothelial cells (ECs) prevent vascular leakage during leukocyte TEM. We examine the correlation between neutrophil extravasation and the evoked permeability changes during acute inflammation *in vitro* and *in vivo*. Spatiotemporal RhoA activation during leukocyte crossing is measured using a recently developed RhoA biosensor^19^. In addition, we use fluorescently-tagged Lifeact and Lifeact-EGFP transgenic knock-in mice to investigate endothelial filamentous (F)-actin dynamics in remodelling junctions during neutrophil diapedesis *in vitro* and *in vivo*, respectively. We show that endothelial pore restriction limits vascular leakage during leukocyte extravasation, which is driven by a basolateral actomyosin-based structure that requires local endothelial RhoA activation.

## Results

### RhoA controls vascular leakage during leukocyte diapedesis

**Figure 1:**
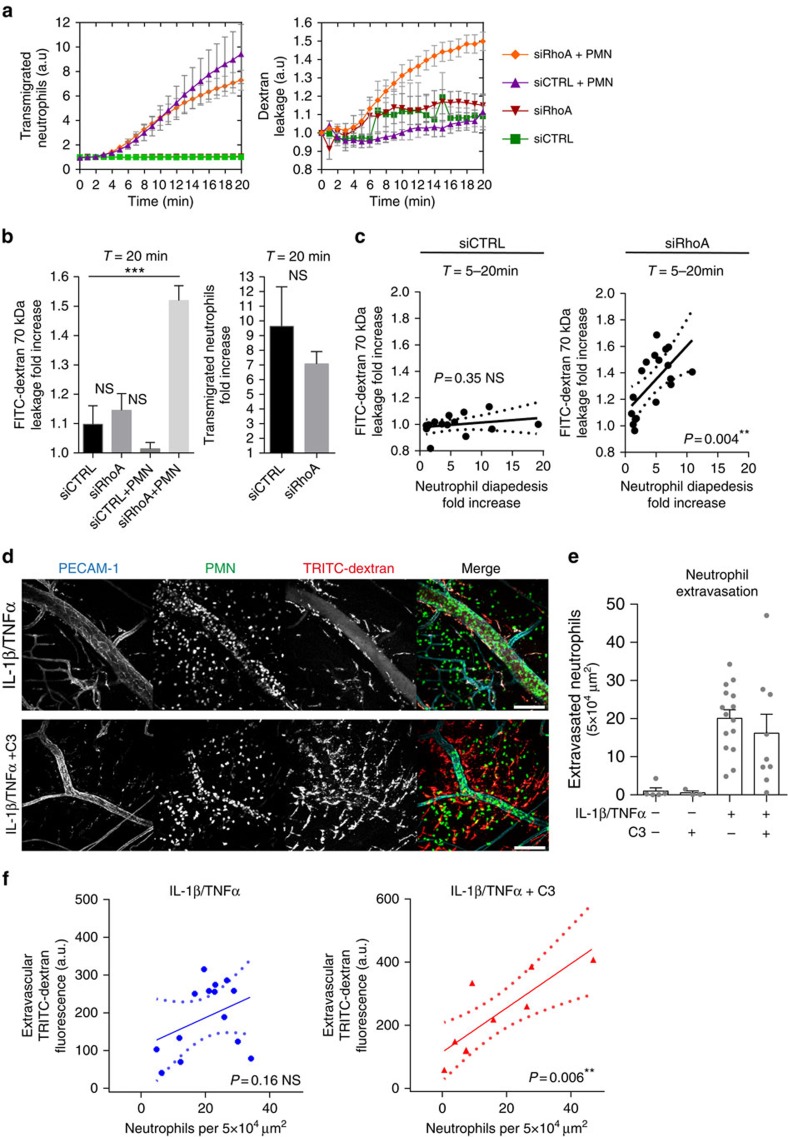
Impaired endothelial RhoA function results in increased vascular leakage during leukocyte diapedesis *in vivo*. (**a**) Extravasation kinetics of calcein-red-labelled neutrophils and FITC–dextran through TNF-α treated ECs cultured on 3-μm pore permeable filtres. Neutrophils transmigrated towards a C5a chemotactic gradient in the lower compartment. Four conditions were tested; RhoA depletion (EC)+neutrophils (Orange line), control+neutrophils (purple line), RhoA depletion (EC) only (red line) and control only (green line). (**b**) Quantification of FITC–dextran and neutrophil extravasation after 20 min of neutrophil transmigration. Immunoblot of RhoA silencing can be found in supplementary fig. 8. (**c**) Correlation analysis of dextran and neutrophil extravasation kinetics through control and RhoA-depleted ECs. (**d**) Confocal intravital microscopy of 20–80 μm diameter cremasteric venulesin LysM–GFP mice (green neutrophils) immunostained *in vivo* for EC junctions by intrascrotal injections of fluorescent-labelled PECAM-1 (blue) and stimulated for four hours with IL-1β and TNF-α only, or with Rho-inhibitor (C3). A second dose of Rho inhibitor was given intrascrotally and TRITC–dextran (40 kDa) was injected intravenously at *T*=2 h and allowed to circulate until *T*=4 h. Scale bar, 100 μm. (**e**) Neutrophil extravasation in animals left unstimulated (control), stimulated with C3 alone, IL-1β/TNFα treated, IL-1β/TNFα treated+C3 or IL-1β/TNFα treated+neutrophil depletion. (**f**) Correlation analysis of dextran and neutrophil extravasation kinetics in animals stimulated with IL-1β/TNFα alone or with IL-1β/TNFα treated+C3. ***P<0.001 control versus RhoA-depleted HUVEC (ANOVA) or *P*=0.3504 control versus RhoA-depleted HUVEC (Student's t-test) (**b**). *r*=0.2547 *P*=0.359 (Pearson's correlation) transmigrated neutrophils versus FITC–dextran leakage in control HUVECs or *r*=0.6345 **P<0.01 (Pearson correlation) transmigrated neutrophils versus FITC–dextran leakage in RhoA-depleted HUVECs (**c**). *P*=0.4230 IL-1β/TNFα versus IL-1β/TNFα+C3 (Student's *t*-test) (**e**). *r*=0.8258 **P<0.01 (Pearson's correlation) transmigrated neutrophils versus FITC–dextran leakage in IL-1β/TNFα+Rho inhibitor treated mice (**f**). Data are from three experiments (**a**–**c**) or are representative of 5 to 13 (**d**–**e**) or 9 (**f**) independent experiments ((**d**–**f**) one mouse per experiment; error bars (**a**–**c**,**e**,**f**), s.e.m).

To investigate the molecular mechanism that controls endothelial barrier function during neutrophil TEM, we simultaneously measured neutrophil transmigration kinetics and fluorescein isothiocyanate (FITC)–dextran leakage across TNFα-stimulated human umbilical vein endothelial cells (ECs) towards a C5a gradient, for 60 min. Neutrophil transmigration across control ECs was associated with minimal FITC–dextran leakage (Fig. 1a). Increasing neutrophil numbers in the upper compartment up to 10-fold did not induce FITC–dextran leakage, indicating that ECs maintained their barrier function, despite increased numbers of transmigrating neutrophils (Supplementary Fig. 1a). To investigate the functional role of RhoA in EC barrier maintenance during neutrophil TEM, we depleted RhoA using siRNA (Supplementary Fig. 1b). We found that endothelial RhoA depletion increased FITC–dextran leakage during neutrophil extravasation, whereas minimal FITC–dextran leakage was measured during neutrophil crossing through control ECs (Fig. 1a,b). Correlation analysis showed that the increase in FITC–dextran leakage was highly correlated to neutrophil transmigration (Fig. 1c). Note that endothelial RhoA depletion did not alter FITC–dextran leakage under basal conditions, which was comparable to control EC (Fig. 1a,b). Moreover, endothelial resistance measured under physiological flow conditions was significantly reduced during transmigration of neutrophils across Rho-inhibited endothelium (Supplementary Fig. 1c). We next investigated the role of RhoA in EC barrier maintenance during neutrophil TEM *in vivo*. Vessel permeability was monitored by Tetramethylrhodamine (TRITC)–dextran leakage into the cremaster of C57BL/6 wild type (WT) or LysM–GFP mice during interleukin (IL)-1β and TNF-α-stimulated neutrophil recruitment. Intrascrotal administration of anti-PECAM-1 labelling antibody resulted in a strong labelling of EC junctions in cremasteric venules (Fig. 1d). Administration of IL-1β and TNF-α enhanced leakage of intravenous TRITC–dextran into the interstitium and neutrophil recruitment into the cremaster (Fig. 1d; Supplementary Fig. 1d). Rho inhibitor I (C3)-treated animals showed similar extravasated neutrophil levels, however, TRITC–dextran leakage in those animals was highly increased compared with IL-1β and TNF-α administration alone (Fig. 1d,e). Although no change in neutrophil extravasation was measured in the presence or absence of C3, we cannot exclude that the inhibitor affects other cells. Neutrophil extravasation and TRITC–dextran leakage in WT mice were not correlated in individual mice, although there was an overall association between extravasation and permeability, whereas the two processes in Rho-inhibited animals showed a highly significant correlation (Fig. 1f). Animals treated with C3 alone showed unaltered basal vascular permeability (Supplementary Fig. 1e). Thus, neutrophil extravasation and evoked changes in vascular permeability during inflammation are not correlated. However, when endothelial RhoA is inhibited, neutrophil diapedesis provokes vascular leakage, suggesting that endothelial RhoA is required to maintain a tight EC barrier during leukocyte diapedesis *in vivo*.

### Spatiotemporal RhoA activation during leukocyte diapedesis

**Figure 2:**
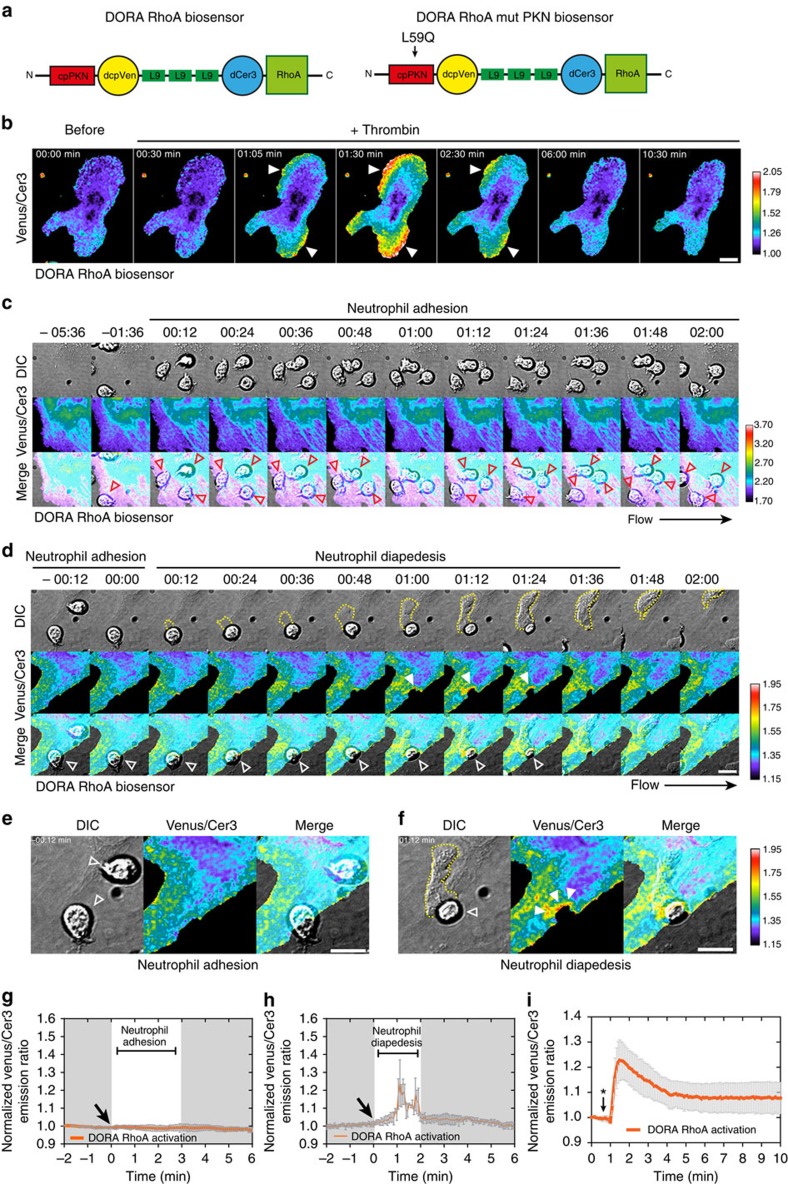
Spatiotemporal RhoA activation during neutrophil TEM. Endothelial RhoA is locally and transiently activated during neutrophil extravasation (**a**) Schematic illustration of the DORA RhoA sensor design containing Rho effector PKN (red), circular permutated Venus (yellow), structured linker protein L9 (green), circular permutated Cer3 (blue) and RhoA GTPase (green),left panel. Right panel shows the DORA RhoA mutant PKN biosensor that was developed as a negative control biosensor, the glutamine was substituted for the leucine at position 59 in the PKN domain. This mutation prevents binding of PKN to activated RhoA. (**b**) Time-lapse Venus/Cer3 ratio images of DORA RhoA biosensor simultaneously recorded with an epi-fluorescent microscope showing spatiotemporal RhoA activation upon thrombin treatment (1 U ml^−1^) in HUVECs. Filled arrows indicate RhoA activation. Scale bar, 10 μm. Calibration bar shows RhoA activation (Red) relative to basal RhoA activity (Blue). (**c**) Epi-fluorescent live-cell imaging of HUVEC expressing the DORA RhoA biosensor during neutrophil adhesion under physiological flow conditions (0.8 dyne per cm^2^). Red open arrows indicate adherent neutrophils. Scale bar, 10 μm. Calibration bar shows RhoA activation (red) relative to basal RhoA activity (blue). (**d**) Epi-fluorescent live-cell imaging of HUVECs expressing the DORA RhoA biosensor during neutrophil TEM. Time-lapse images of DIC (upper) Venus/Cer3 ratio images of DORA RhoA biosensor (middle) and Merge (bottom) during leukocyte diapedesis. Open arrows indicates adherent neutrophil at the apical side of the endothelium. Filled arrows indicate local RhoA activation during neutrophil diapedesis. Scale bar, 10 μm. (**e**) Detailed zoom of RhoA activation during neutrophil adhesion (open arrows) prior diapedesis at time point *t*=−00:12 min. (**f**) Detailed zoom of local RhoA activation during neutrophil transmigration at time point *t*=01:12 min. Filled arrows indicate local RhoA activation during neutrophil diapedesis. Scale bar, 10 μm. (**g**) Quantification of temporal RhoA activation during multiple neutrophil transmigration events starting at time zero (arrow). (**h**) Quantification of temporal RhoA activation during multiple neutrophil adhesion events starting at time zero (arrow). (**i**) Quantification of DORA RhoA biosensor activation after thrombin treatment (1 U ml^−1^) in HUVEC. Asterisk indicates thrombin addition. Data represent mean and s.e.m of 7 experiments (**g**) 5 experiments and (**h**) 10 experiments (**i**).

To examine spatiotemporal RhoA activation in ECs during EC barrier maintenance associated with neutrophil TEM, we used a recently developed fluorescence resonance energy transfer (FRET)-based RhoA biosensors called the Dimerization Optimized Reporter for Activation (DORA) RhoA sensors (Fig. 2a)^19^. DORA RhoA biosensors design were based on the published RhoA biosensor^20^. The ON-state FRET efficiency of the GTPase was improved through modelling of the fluorescent protein dimers and the GTPase-effector domain complexes. Stable α-helical repeats from ribosomal protein L9, rather than an unstructured linker, were inserted between the fluorescent proteins to disrupt dimerization and diminish FRET efficiency in the inactive state (Fig. 2a). As a control, DORA RhoA mutant Protein kinase N (PKN) was developed to report misalignment of Cerulean3 (Cer3) and Venus image before and after image registration, motion artefacts or pH changes affecting the sensors fluorescent proteins. Glutamine substitution for a leucine at position 59 in the PKN domain prevents PKN binding to activated RhoA^21^. The characterizations of both DORA RhoA biosensors are described in online methods (Supplementary Figs 2 and 3a). From these validation experiments, we conclude that the DORA RhoA biosensor accurately reports RhoA dynamics in ECs downstream from endogenous stimuli such as thrombin (Fig. 2b; Supplementary Fig. 1f).

To study spatiotemporal RhoA activation in ECs during neutrophil TEM, we expressed the DORA RhoA biosensor in ECs and investigated RhoA activation following neutrophil extravasation under physiological flow conditions. Important to note, Venus and Cer3 emission were simultaneously recorded utilizing a double-camera system, since sequential image acquisition resulted in motion artefacts induced by migrating leukocytes displacing fluorescent signals in ECs. We found unaltered RhoA biosensor activation during neutrophil rolling and crawling over the endothelium (Fig. 2c,g). Also RhoA activation during the initial opening of EC junctions was found to be unaltered (Fig. 2d). However, RhoA biosensor activity in the endothelium was locally increased at sites were neutrophils breeched the endothelium, between the first and second minute of neutrophil diapedesis (Fig. 2d–f, Supplementary Fig. 2d; Supplementary Movie 1). On the basis of the normalized ratiometric imaging and the relative displacement of the sensor, the data showed a 1.2-fold increase in FRET ratio on diapedesis, comparable to what has been observed during RhoA activation after thrombin stimulation (Fig. 2h,i). The negative control DORA RhoA biosensor (mutant PKN) showed no change in FRET during leukocyte diapedesis (Supplementary Fig. 3b,c; Supplementary Movie 2). Importantly, expressing the DORA RhoA biosensors in ECs did not interfere with neutrophil TEM. Thus, endothelial RhoA is transiently and locally activated during the final stage of neutrophil diapedesis, but not during crawling or opening of endothelial junctions, indicating a role for local RhoA activity in EC barrier maintenance during the final stage of neutrophil extravasation.

### F-actin-rich endothelial pores during diapedesis

**Figure 3:**
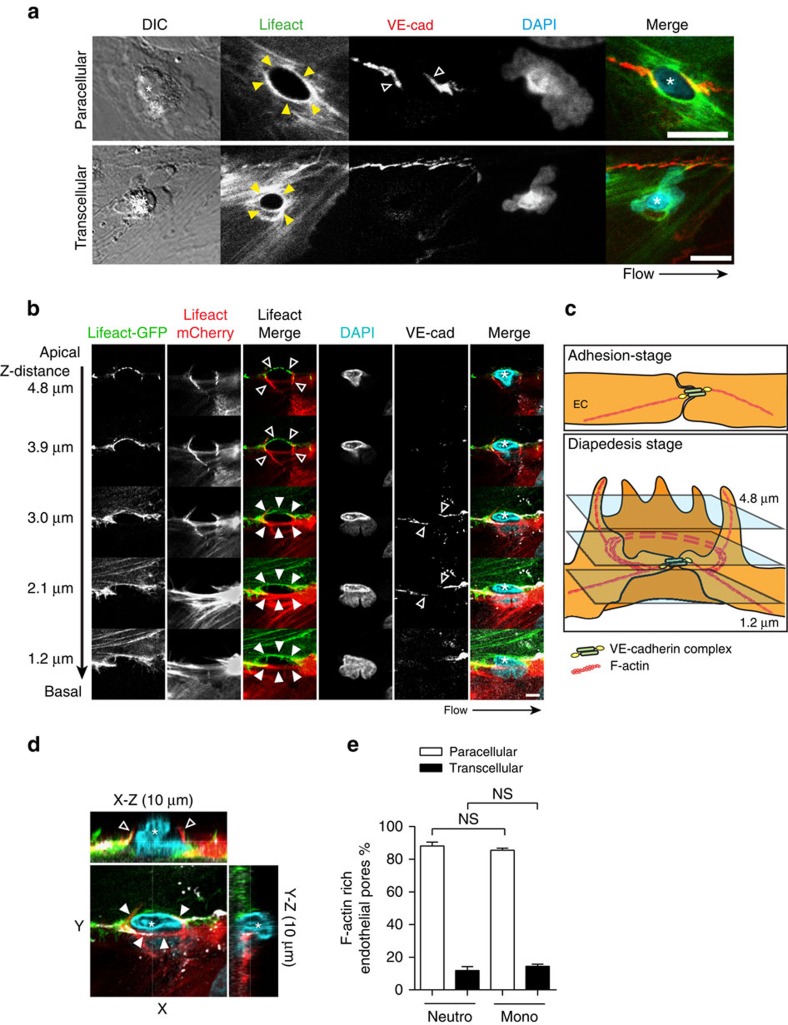
ECs assemble F-actin-rich ring-like structures around transmigrating leukocytes. (**a**) Confocal imaging of para- and transcellular migrating leukocytes through Lifeact-GFP expressing HUVECs. Filled arrows indicate EC F-actin (Lifeact in green) assembly around extravasating leukocytes. Open arrows indicate VE-cadherin (directly labelled Alexa-647 antibody in red) distribution to the F-actin structure sites during paracellular diapedesis. Asterisks indicate extravasating leukocyte (DAPI in blue) in DIC. Flow speed 0.8 dyne per cm^2^. Scale bar, 5 μm. (**b**) Confocal imaging showing a Z-stack of Lifeact-GFP and Lifeact-mCherry positive endothelial membrane structures from apical to basal plane. Open arrows indicate filopodia-like protrusions at the apical site of the structure. Filled arrows indicate the cortical actin ring at the basolateral site that appeared during leukocyte crossing. Asterisk indicates extravasating leukocyte (DAPI in blue). Scale bar, 5 μm. (**c**) Cartoon of endothelium during basal-stage and leukocytes diapedesis showing filopodia-like protrusions and the basolateral F-actin ring. (**d**) *X*–*Z* (10 μm) and *Y*–*Z* (10 μm) projections of confocal Z-stack shown in Fig. 1b. (**e**) Quantification of percentage F-actin-rich endothelial pores associated with neutrophil and monocyte extravasation during para- and transcellular migration. Statistical significance was tested with ANOVA. Data are representative for three independent experiments (**a**–**e**) with 37–65 transmigration events per group (error bars (**e**) s.e.m).

To investigate endothelial F-actin dynamics during neutrophil diapedesis, we transfected ECs with GFP- and/or mCherry-tagged Lifeact^22^. It is important to note that phalloidin staining to visualize F-actin cannot be used to investigate endothelial actin structures that are in close proximity of transmigrating leukocytes, since F-actin in both leukocytes and ECs are visualized by phalloidin staining, making it impossible to discriminate between the two (Supplementary Fig. 3d). Transmigrating neutrophils initiated small endothelial pores in the endothelial lining. To study those endothelial pores at high resolution, transmigrating neutrophils were fixed with formaldehyde when partly breeched the endothelium. Confocal microscopy imaging and three-dimensional (3D) reconstruction showed that ECs assembled F-actin-rich structures around endothelial pores through, which neutrophils transmigrated, both during transcellular and paracellular migration (Fig. 3a; Supplementary Movies 3–5). During paracellular migration, the junctional protein VE-cadherin was distributed to the endothelial pore margins (Fig. 3a). Interestingly, using ECs expressing either Lifeact-GFP or Lifeact-mCherry, we found that paracellular pores were formed by at least two ECs. At the structures apical site, filopodia-like protrusions were found, whereas at the basolateral site, a cortical actin ring appeared during leukocyte crossing (Fig. 3b–d). In contrast to VE-cadherin distributed to the pores margins, the junctional protein PECAM-1 was localized around the basolateral F-actin ring and distributed to apical protrusions surrounding migrating leukocytes during trans-and paracellular migration. (Supplementary Fig. 3e,f). Moreover, we found that the adhesion molecule ICAM-1 was localized in the apical protrusions at endothelial pores (Supplementary Fig. 4a). We found that ∼90% of all neutrophils and monocytes used the paracellular route, whereas ∼10% migrated transcellular, in line with the migratory preference for neutrophils and monocytes found *in vivo*^23^ (Fig. 3e). Note that all these diapedesis events by either neutrophils or monocytes were associated with basolateral F-actin ring formation around endothelial pores (Fig. 3e). Within the paracellular route of migration, leukocyte transmigration through a bi-cellular or a multicellular junction was ∼50% (Supplementary Fig. 4b). In conclusion, ECs assemble F-actin-rich ring-like structures around endothelial pores through which neutrophils and monocytes transmigrate. This data indicate that maintenance of EC barrier function during leukocyte diapedesis involves actin cytoskeleton strengthening around endothelial pores. Basolateral F-actin ring formation may tighten the endothelial barrier during neutrophil crossing, making the leukocyte-induced endothelial pore impermeable for macromolecules.

### F-actin-rich endothelial pores are confined in size

Electron microscopy studies showed that ECs maintain intimate contact with transmigrating neutrophils during the entire transmigration process^15^,^24^. To examine the dynamic contact between ECs and extravasating neutrophils, we examined F-actin-enriched endothelial pore shape and size in relation to neutrophil size. Real-time recordings of transmigrating neutrophils through ECs expressing GFP-tagged Lifeact showed increased F-actin assembly around endothelial pores (Supplementary Movie 6).

**Figure 4:**
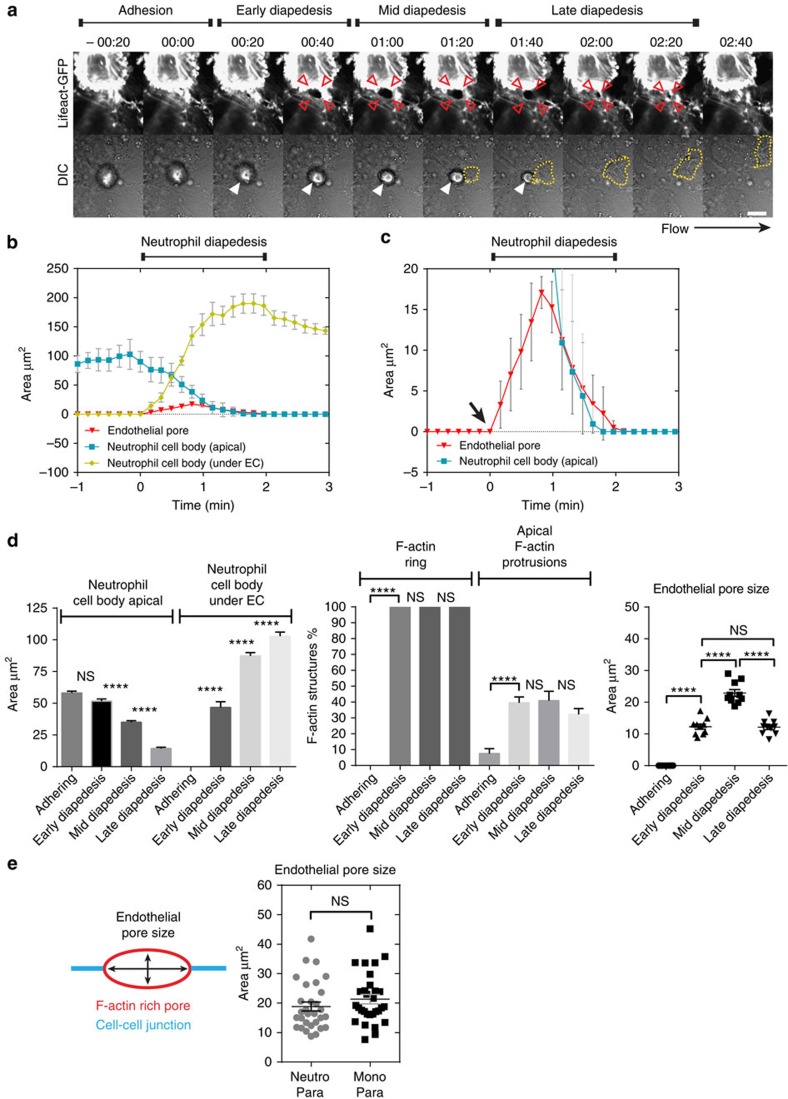
Endothelial pores formed during para- and transcellular leukocyte transmigration are confined in size. (**a**) Epi-fluorescent live-cell imaging of ECs expressing Lifeact-GFP. Red open arrows indicate F-actin-rich endothelial pore formation during leukocyte diapedesis under physiological flow conditions (0.8 dyne per cm^2^). Filled arrows indicate extravasating leukocyte in DIC. Dashed lines indicates neutrophil localization under the endothelium. Scale bar, 10 μm. (**b**,**c**) Quantification of size changes occurring in the neutrophil cell body and endothelial pore during neutrophil diapedesis. Endothelial pore size (red), neutrophil cell body apical (blue) and neutrophil cell body under EC (yellow), diapedesis starts at time zero. (**d**) Quantification of Neutrophil size, F-actin-positive ring structures, F-actin positive apical protrusions and endothelial pore size. (**e**) Quantification of endothelial pore size for neutrophils and monocytes during paracellular migration. ****P<0.0001 (analysis of variance). Data are representative of four independent experiments (**d**,**e**) with 40 transmigration events per group. Data in **b** and **c** are representative of 10 transmigration events (error bars (**b**–**e**) s.e.m).

The kinetics of neutrophil diapedesis is on an average 2 min and can be distinguished into early, mid and late diapedesis based on endothelial pore size and neutrophil morphology (Fig. 4a–c). Endothelial pore formation started when neutrophils partly breeched the endothelium, defined as early diapedesis. Following neutrophil diapedesis, most endothelial pores are maximal enlarged 1 min after transmigration was initiated, defined as mid diapedesis (Fig. 4a–c). Subsequently, the endothelial pore is closed in conjunction with transmigrating neutrophils until completely under the endothelium, a stage defined as late diapedesis (Fig. 4a–c). Real-time imaging of neutrophil diapedesis under physiological flow conditions showed that neutrophil total surface area before TEM was roughly 100 μm^2^, which was reduced to <20 μm^2^ to fit the confined gap in the endothelium that had a maximal inner-surface area of 19 μm^2^ (Fig. 4b,c). To investigate the morphology of *de novo* formed F-actin-positive rings and F-actin-positive apical protrusions that surround endothelial pores during neutrophil TEM, we trapped neutrophils at different stages of diapedesis. Interestingly, *de novo* formed F-actin-positive rings surrounding endothelial pores were found throughout all diapedesis steps, but not during neutrophil adhesion or crawling steps (Fig. 4d; Supplementary Fig. 4c). Quantification of endothelial pore size showed significant larger pores during mid diapedesis than during early and late diapedesis when pores open and close, respectively (Fig. 4d). We next measured, the pore size width, length and height of F-actin-rich endothelial pores surrounding transmigrating neutrophils and monocytes. On average, endothelial pores are 4-μm wide, 6-μm in length and mostly oval shaped for all leukocytes migrating through the cell–cell junctions (Supplementary Fig. 4d,f). In addition, we found that only during diapedesis ∼40% of the endothelial pores contained F-actin-rich apical protrusions (Fig. 4d). No such structures were detected during the crawling step. These structures reached a maximal height of 6–7 μm (Supplementary Fig. 4e). Transcellular pores were found to be more round or circular shaped and had an average circularity of about 1.3 according to the circularity index (Supplementary Fig. 4f). Endothelial pore sizes showed remarkably little variation, despite leukocyte size, type or transmigration route (Fig. 4e). Thus, endothelial pores induced by extravasating neutrophils and monocytes are confined in size and close directly behind transmigrated cells. Active endothelial pore confinement and pore closure corroborated earlier findings that showed intimate contact between neutrophils and ECs during the entire TEM process and provides an explanation for limited transendothelial escape of macromolecules during neutrophil crossing.

### Pore confinement and pore closure requires endothelial RhoA

**Figure 5:**
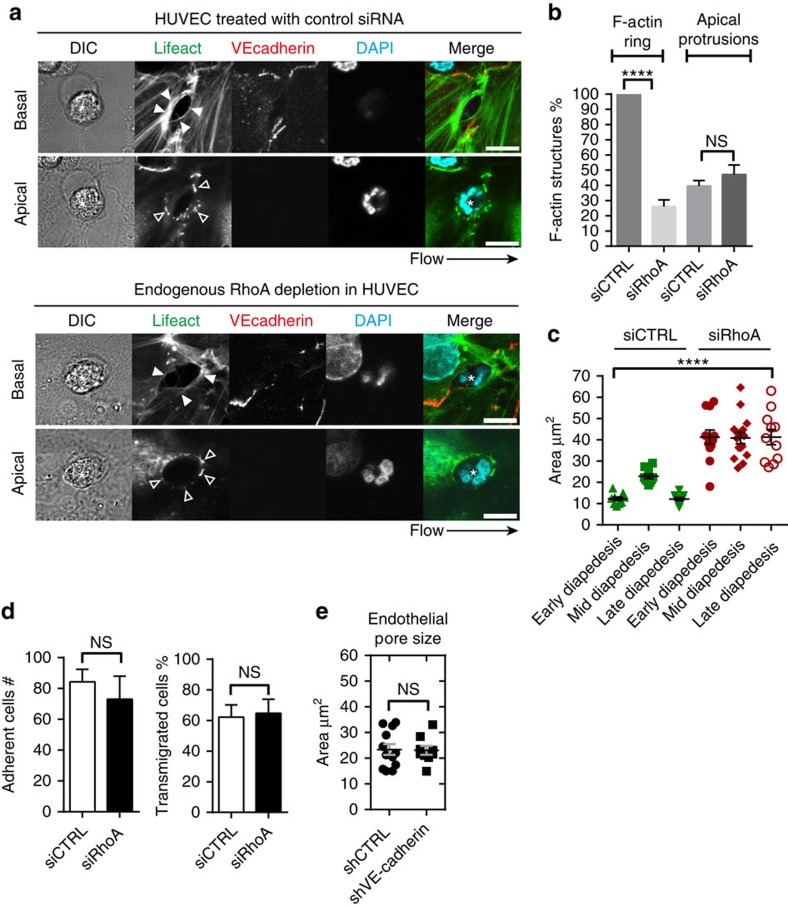
RhoA signalling is required for endothelial pore confinement. (**a**) Confocal imaging of paracellular migrating neutrophils through Lifeact-GFP expressing HUVECs after 72-h transfection with control siRNA (upper panel) or RhoA siRNA (lower panel) under physiological flow conditions (0.8 dyne per cm^2^). Open arrows and filled arrows indicate filopodia-like protrusions at the apical site and the cortical F-actin ring at the basolateral site of the endothelial pore, respectively. Asterisk indicates extravasating neutrophil (DAPI in blue). VE-cadherin (red). Scale bar, 5 μm. (**b**) Quantification of F-actin-positive ring structures and F-actin-positive apical protrusions in control versus RhoA-depleted ECs. (**c**) Quantification of endothelial pore size during early, mid and late diapedesis. (**d**) Quantification of neutrophil adhesion and diapedesis through TNF-α treated ECs under physiological flow conditions after 72 h transfection with control siRNA (open bar) or RhoA siRNA (filled bar). (**e**) Quantification of endothelial pore size in control versus VE-cadherin depleted HUVECs. ****P<0.0001 (analysis of variance). Data are representative of four independent experiments (**a**–**e**) with >12 transmigration events per group (error bars (**c**,**e**) s.e.m).

Our data showed that increased endothelial RhoA activity during neutrophil TEM corresponded to endothelial pore restriction and closure during mid and late diapedesis. To investigate whether RhoA regulates endothelial pore confinement, we silenced endothelial RhoA using siRNA. RhoA was successfully depleted as shown by western blot analysis (Supplementary Fig. 5a). Confocal microscopy showed that RhoA depletion in ECs reduced Lifeact-GFP accumulation around endothelial pores, whereas Lifeact-GFP in the apical protrusions was still present (Fig. 5a). Basal F-actin rings in RhoA-depleted ECs were significantly reduced compared to control conditions (Fig. 5b). Endothelial RhoA depletion had no effect on the formation of F-actin-rich apical protrusions (Fig. 5b). Quantification of endothelial pore size showed that in the absence of RhoA, endothelial pores were not only larger than endothelial pores formed in control ECs but also did not close properly (Fig. 5c). Note that neutrophil adhesion and transmigration under physiological flow conditions were unaltered in RhoA-depleted ECs (Fig. 5d). To study if VE-cadherin signalling regulates endothelial pore size, we depleted VE-cadherin and analysed endothelial pore size. However, VE-cadherin depletion had no effect on endothelial pore size (Fig. 5e; Supplementary Fig. 5b–d). In conclusion, RhoA facilitates endothelial pore confinement and pore closure during leukocyte diapedesis.

### Pore confinement is driven by actomyosin contractility

**Figure 6:**
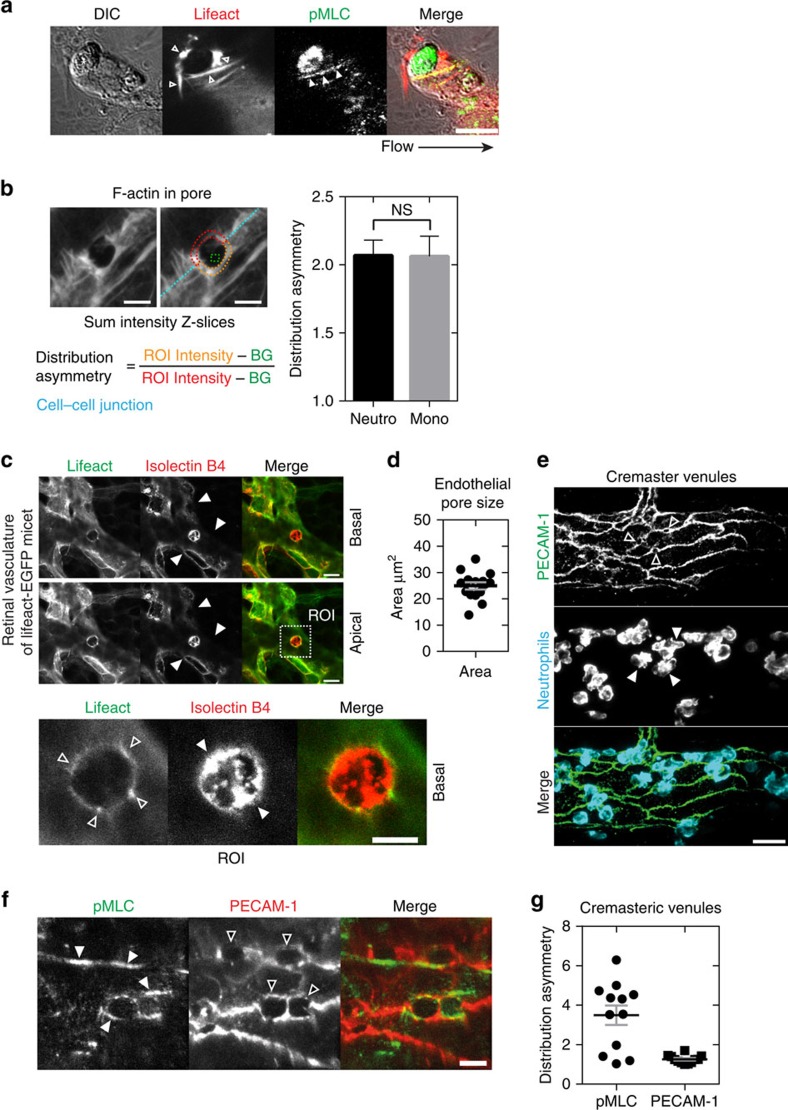
Endothelial pore confinement is driven by actomyosin contractility. (**a**) Immunofluorescence analyses of MLC phosphorylation during neutrophil transmigration. Open and filled arrows indicate Lifeact-mCherry (red) and MLC phosphorylation (green) localization, respectively, during neutrophil transmigration under physiological flow conditions (0.8 dyne per cm^2^). Asterisk indicates extravasating leukocyte in DIC. Scale bar, 10 μm. (**b**) Quantification of F-actin distribution in endothelial pores surrounding transmigrating neutrophils and monocytes. Maximum intensity projection of F-actin in the endothelial pore was used to quantify F-actin distribution surrounding transmigrating leukocytes. Distribution asymmetry is defined by the ratio of region of interest ROI-1 and ROI-2 corrected for background. Scale bar, 5 μm. (**c**) Confocal imaging of F-actin dynamics during leukocyte diapedesis in retina vasculature of Lifeact-EGFP C57BL6 mice. Filled arrows indicate the vasculature of mice retina, highly expressing Lifeact-GFP. Zoom of ROI, open arrows indicate the Lifeact-EGFP (green)-positive endothelial pore, filled arrows indicate transmigrating neutrophil. Scale bar, 5 μm. (**d**) Quantification of endothelial pore size in retina vasculature. (**e**) Confocal imaging of PECAM-1 in cremasteric venules during TNF-α and IL-1β induced neutrophil recruitment. Open arrows indicate PECAM-1 positive endothelial pores that surround extravasating neutrophils (filled arrows). Scale bar, 20 μm. (**f**) IF analyses of MLC phosphorylation during neutrophil transmigration into the cremaster of C57BL6 mice. Filled and open arrows indicate phospo-MLC and PECAM-1 localization to endothelial pores, respectively. Scale bar, 5 μm. (**g**) Quantification of pMLC and PECAM-1 localization in endothelial pores. We quantified MLC phosphorylation defined as distribution asymmetry. The distribution asymmetry uses the intensity of one ROI versus another ROI as indicated in **b**. Because MLC may occur at different heights within the pore we used max projection for this analysis. Data are representative of three independent experiments (**a**–**g**) with >12 transmigration events per group (error bars (**b**,**d**,**g**) s.e.m).

To investigate how RhoA regulates endothelial pore confinement during leukocyte diapedesis, we examined RhoA effector myosin II activation. To study myosin II activation we locally measured myosin light-chain (MLC) phosphorylation on position Thr18 and Ser19. Immunofluorescent staining of pMLC showed an asymmetric phosphorylation pattern in F-actin-rich endothelial pores surrounding transmigrating neutrophils (Fig. 6a). MLC was particularly phosphorylated at cortical actin bundles as part of the F-actin ring (Fig. 6a; Supplementary Fig. 5e). Note that the uropod of the neutrophil is positive for MLC phosphorylation, most likely to retract its tail during transmigration^25^. In contrast to local MLC phosphorylation in control ECs, endothelial pores in RhoA-deficient ECs were enlarged and negative for local phospho-MLC (Supplementary Fig. 5e). In addition, we quantified Lifeact-GFP distribution around endothelial pores and found asymmetric F-actin distribution around endothelial pores, indicative of increased tension (Fig. 6b). To corroborate our findings *in vivo*, we studied F-actin localization during leukocyte diapedesis in retinal vasculature of Lifeact-EGFP-transgenic knock-in mice^26^. Lifeact-EGFP expression in the retinas of these mice is largely restricted to the endothelium and this allowed us to properly visualize F-actin in ECs *in situ*^26^,^27^. We found that endothelial pores induced by transmigrating neutrophils (isolectin B4-positive^28^) were surrounded by Lifeact-EGFP-positive rings in retinal ECs (Fig. 6c). Quantification of these rings showed that endothelial pore size *in vivo* was comparable to endothelial pores measured in the *in vitro* set-up (compare Figs 6d and 4e). Lifeact was present in the basolateral ring and in apical protrusions that surrounded transmigrating neutrophils (Supplementary Fig. 5f). These data showed that apical membrane protrusions *in vivo* are rich for F-actin and surround adherent leukocytes. Next, we examined local MLC phosphorylation in WT mice during IL-1β and TNF-α-induced neutrophil recruitment in cremasteric venules. PECAM-1 was used as a marker to visualize endothelial pores *in vivo*^23^ (Fig. 6e). In line with our *in vitro* findings, endothelial pores in mouse cremaster venules showed asymmetric MLC phosphorylation (Fig. 6f,g). To address the role of ROCK in endothelial pore confinement we depleted the ROCK isoforms ROCK1 and ROCK2b in endothelial cells and examined vascular permeability during neutrophil diapedesis. In line with RhoA inhibition, silencing ROCK 1 and ROCK2b did not prevent the adhesion or transmigration of neutrophils through the endothelial monolayer (Supplementary Fig. 6a,b). Basal endothelial barrier function in ROCK1 or ROCK2b deficient ECs was not affected. However, neutrophil diapedesis through ROCK2b, but not ROCK1 deficient ECs elicited increased endothelial permeability up to a twofold (Supplementary Fig. 6a,b). These findings may indicate that endothelial pore confinement is mediated through ROCK2b but not ROCK1. Altogether, local accumulation of F-actin and MLC phosphorylation is associated with neutrophil diapedesis *in vitro* and *in vivo,* suggesting that endothelial pore confinement is driven by local actomyosin contractility.

### Pore confinement requires ICAM-1, LARG and Ect2

**Figure 7:**
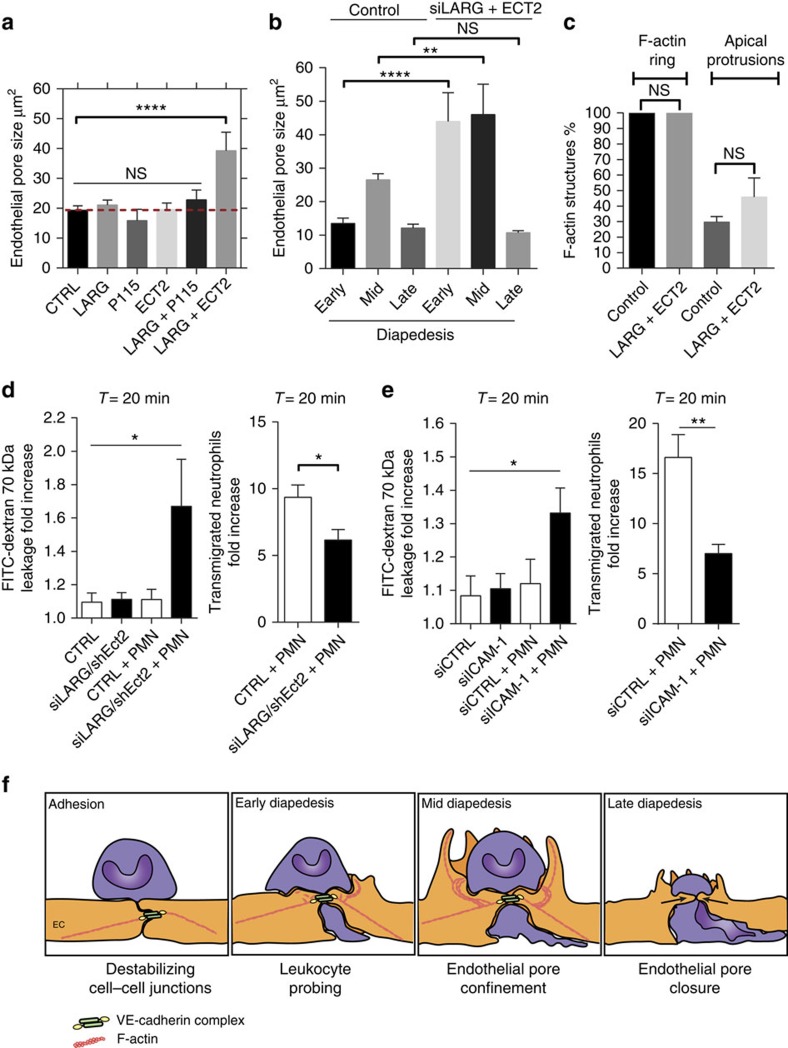
ICAM-1 regulates endothelial pore confinement through recruitment of the Rho GEFs LARG and Ect2. (**a**) Quantification of endothelial pore size in LARG, p115RhoGEF or Ect2 depleted ECs. (**b**) Quantification of endothelial pore size during early, mid and late diapedesis in control versus LARG+Ect2 depleted ECs. (**c**) Quantification of F-actin-positive ring structures and F-actin-positive apical protrusions in control versus LARG+Ect2 depleted ECs.(**d**) Quantification of FITC–dextran and neutrophil extravasation after 20 min of neutrophil transmigration through control and Ect2/LARG (**d**) or ICAM-1-deficient ECs (**e**). (**f**) Model of endothelial pore formation. Adherent leukocytes destabilizing cell–cell junctions subsequently insert small pseudopodia between transient openings in the endothelium (leukocyte probing) during early diapedesis. The next step (mid diapedesis) involves local and transient RhoA activation that mediates endothelial pore confinement through the formation of a *de novo* basolateral F-actin ring and actomyosin contractility. Finally, persistent actomyosin contractility closes the endothelial pore behind transmigrating leukocytes. ICAM-1 is involved in the regulation of endothelial pore confinement through recruitment of the Rho GEFs LARG and Ect2. Basolateral F-actin ring formation and actomyosin contractility tightens the endothelial barrier during leukocyte diapedesis, making the leukocyte-induced endothelial pore impermeable for macromolecules. ****P<0.0001 (ANOVA) (**a**–**c**).**P<0.01 (ANOVA) (**b**) *P<0.05 (ANOVA) (**e**,**d**).*P<0.05 control versus siLARG/shEct2 (**d**) **P<0.01 control versus siICAM-1 (**e**) (Student's *t*-test). Data are representative of three independent experiments (**a**–**e**) with >12 transmigration events per group (**a**–**c**) (error bars (**a**–**e**) s.e.m). ANOVA, analysis of variance.

To investigate the signalling events upstream from RhoA, we focused on the involvement of guanine-nucleotide exchange factors (GEF) and performed a GEF screen that included: p115RhoGEF, Ect2 and LARG. Depletion of endothelial LARG together with Ect2 increased endothelial pore size, whereas depletion of LARG, p115 and Ect2 alone was not sufficient (Fig. 7a; Supplementary Fig. 6c). Quantification of endothelial pore size at different stages of diapedesis showed that endothelial pores in LARG- and Ect2-depleted endothelium were enlarged during early and mid diapedesis, but had no effect on endothelial pore closure (Fig. 7b). Under these conditions the number of F-actin-positive rings and F-actin-positive apical protrusions was unaltered (Fig. 7c). Enlarged endothelial pores in Ect2- and LARG-deficient ECs showed increased endothelial permeability during neutrophil diapedesis, whereas basal EC barrier function was not affected (Fig. 7d; Supplementary Fig. 6d–f). Neutrophil diapedesis through Ect2- and LARG-deficient ECs was slightly reduced (Fig. 7d; Supplementary Fig. 6d). To study LARG and Ect2 recruitment to the intracellular tail of PECAM-1 or ICAM-1 we performed clustering experiments induced by anti-ICAM-1- or anti-PECAM-1-coated beads. We found that LARG and Ect2 are recruited to the intracellular tail of ICAM-1 (Supplementary Fig. 6g).Whereas PECAM-1 recruited only LARG, but not Ect2 to its intracellular tail on clustering (Supplementary Fig. 6h). To investigate whether ICAM-1 or PECAM-1 initiate and coordinate local RhoA activation and endothelial pore confinement during neutrophil diapedesis, we depleted ICAM-1 and PECAM-1 in ECs and examined the extravasation of calcein-red-labelled neutrophils and FITC–dextran across EC and measured endothelial pore size. We found that neutrophil transmigration through ICAM-1-deficient ECs compromised the endothelial barrier (Fig. 7e; Supplementary Fig. 7a–c), whereas PECAM-1 depletion did not alter endothelial pore size or vascular leakage (Supplementary Fig. 7d–e). ICAM-1 and PECAM-1 depletion alone had no effect on endothelial permeability (Fig. 7e; Supplementary Fig. 7d). In agreement with the existing literature, endothelial ICAM-1 depletion significantly reduced the number of transmigrated neutrophils (Fig. 7e). Neutrophil diapedesis through PECAM-1-deficient ECs showed no reduction in transmigration numbers (Supplementary Fig. 7d). These data point out an important role for ICAM-1, Ect2 and LARG signalling in controlling RhoA-mediated endothelial pore confinement and EC barrier protection during neutrophil diapedesis.

## Discussion

Leukocytes that cross the endothelium induce large endothelial gaps without provoking leakage of plasma into the underlying tissue. However, the mechanisms behind this intriguing phenomenon are unclear. The present study shows how ECs limit vascular leakage during leukocyte TEM. We found that RhoA-mediated F-actin rings contribute to endothelial pore confinement that maintains endothelial barrier integrity during leukocyte diapedesis. Neutrophil diapedesis through ICAM-1-, Ect2/LARG- and RhoA-deficient ECs provokes vascular leakage that was highly correlated with neutrophil breeching events. Mechanistically, we found that the initiation of RhoA activity involves ICAM-1 and the Rho GEFs Ect2 and LARG. In addition, we found that the regulation of actomyosin-based endothelial pore confinement involves ROCK2b, but not ROCK1. Our work identifies a novel mechanism that maintains endothelial barrier integrity during leukocyte extravasation, which is driven by a basolateral actomyosin-based structure that requires spatiotemporal RhoA cycling (Fig. 7f).

Inflammation-driven leukocyte recruitment and vascular permeability are separable events^7^,^8^,^17^. In line with these observations, we discovered that during the TEM process endothelial RhoA plays a central role in EC barrier maintenance, but is redundant for leukocyte transmigration. In agreement with previous reports, blocking RhoA activity or depleting RhoA in ECs did not perturb adhesion^29^ or transmigration^30^. In contrast to the general concept that RhoA activation is required for leukocyte adhesion and opening of endothelial junctions^31^,^32^,^33^,^34^,^35^, we found that endothelial RhoA was locally and transiently activated during the diapedesis step and not during neutrophil crawling, firm adhesion or opening of endothelial junctions prior to leukocyte extravasation. These processes require a separate, RhoA-independent mechanism that allows leukocyte–EC adhesion or opening of endothelial junctions. In agreement with our findings, for both transmigration routes, endothelial pore opening is in part mediated by mechanical forces that are generated by migrating leukocytes. Polarized actin polymerization in the leukocyte elicits pulling and pushing forces that support the movement of immune cells through the confined endothelial pore^36^,^37^. ICAM-1 is known to mediate leukocyte–EC interactions, and crosslinking ICAM-1 using ICAM-1-coated beads or ICAM-1 antibodies results in increased RhoA activation suggesting a role for ICAM-1-mediated RhoA activation in leukocyte adhesion^32^,^38^,^39^,^40^,^41^. However, based on the spatiotemporal activation of RhoA, we suggest that ICAM-1-mediated RhoA signalling specifically occurs during the diapedesis step, in agreement with our data that shows ICAM-1 enrichment only at diapedesis sites. PECAM-1 was also detected at sites of diapedesis, for either paracellular or transcellular migration. Recently, mechanical tension exerted on ICAM-1 and also PECAM-1 enhanced RhoA activation and MLC phosphorylation in ECs that was dependent on the recruitment of the RhoGEF LARG and ICAM-1 clustering^41^,^42^. Our work shows that ICAM-1 clustering indeed promotes the recruitment of LARG and we additionally found Ect2 to be recruited on ICAM-1 clustering. Depletion of ICAM-1- and Ect2/LARG in ECs compromised the endothelial barrier during neutrophil diapedesis. Indicating that the ICAM-1-LARG/Ect2 signalling axis is likely to be activated upstream from RhoA activation, to regulate *de novo* F-actin rearrangements, endothelial confinement and barrier protection during neutrophil crossing. Altogether these data suggest that leukocytes exert mechanical forces on endothelial adhesion molecules that modulate endothelial F-actin cytoskeleton through mechanotransduction that may cause endothelial confinement.

The relationship between Ect2 and actomyosin contractility has been clearly established by several studies. For instance, Ect2 has been described to be involved in RhoA activation and contractile ring formation during cytokinesis^43^. In addition, it has been shown that the molecular pathways that regulates local RhoA activation during cytokinesis are also used to control RhoA dynamics at the zonula adherens in interphase cells^44^. Although no proof for a role of Ect2 in endothelial junction regulation has been described we can speculate that Ect2 mediates similar functions in ECs, regulating actomyosin contractility around the pore. The latter is probable, since depletion of LARG and Ect2 simultaneously results in larger pores without affecting the number of F-actin rings. In agreement with this hypothesis overall endothelial pore size in RhoA-deficient endothelial cells was increased due to the lack of basal F-actin ring formation. In addition, we observed that RhoA-deficient endothelial cells were unable to phosphorylate MLC near endothelial pores. Surprisingly, the length-to-width aspect ratio between paracellular and transcellular pores was found to be different; however, this was not due a difference in nuclear size, shape or composition between neutrophils and monocytes. We speculate that in case of paracellular migration the amount of VE-cadherin disassembly at the pores margins may regulate pore size, which may affect the length/width ratio or circularity. In case of transcellular migration mechanical forces from the endothelium might counteract leukocyte-induced forces from all directions forcing a circular passageway. A physical explanation for circular transcellular passages may also explained by cellular dewetting^45^. Despite different length-to-width ratio of the pores, the overall pore size is constant independent of leukocyte type, or transmigration route. This may indicate that the contractile forces generated during endothelial pore formation are high enough to counteract the mechanical forces generated by transmigrating leukocytes. Alternatively, the RhoA-induced basolateral F-actin ring itself may also add as a limiting factor for pore confinement on top of the actomyosin-based contractility. Endothelial pore confinement is probably not restricted to neutrophil and monocyte diapedesis but may also occur during the diapedesis of other immune cells such as T-lymphocytes. Additional research is required to investigate this hypothesis. Surprisingly, we found that VE-cadherin depletion did not affect endothelial pore integrity, despite the prominent VE-cadherin localization at the pores margins. It is known that other junctional molecules such as *N*-cadherin may take over the function of VE-cadherin when VE-cadherin is depleted^46^. The fact that we see unaltered pore morphology in VE-cadherin deficient cells makes it conceivable that other molecules like *N*-cadherin take over the function of VE-cadherin controlling the integrity of the endothelial pore at its margins. It is evident that VE-cadherin plays a dominant role in endothelial barrier formation and regulation of leukocyte traffic through the endothelial barrier. For instance, locking VE-cadherin junctions reduces the number emigrating leukocytes^47^ and the phosphorylation of VE-cadherin at Y731 induced by adherent leukocytes prior diapedesis is a necessity for junctional destabilization and paracellular diapedesis^17^. We cannot exclude a supportive role for VE-cadherin in endothelial pore integrity, but we can exclude a direct role for VE-cadherin as a signalling molecule being involved in controlling and coordinating of endothelial pore confinement. Whether other junctional proteins such as JAM-A or CD99, that act distally from ICAM-1, and signal to RhoA to prevent leakage is currently unknown^23^,^48^. We found that many F-actin rings comprise apical membrane protrusions. These projections, also known as ‘docking structures' or ‘transmigratory cups', have been suggested to anchor endothelial adhesion receptors and therefore control leukocyte adhesion^30^,^39^,^49^,^50^,^51^,^52^. However, the biological function of these structures is still under debate. Interestingly, we found F-actin rings associated with leukocyte diapedesis that contained no apical protrusions suggesting that directional neutrophil diapedesis can occur through other mechanisms than ‘apical projection'-guidance for instance transendothelial migration-promoting endothelial chemokines that are locally released within the endothelial pore^53^. In agreement with studies showing that apical projection assembly requires RhoG and Rac1 but not RhoA activity^39^,^54^, we still observed apical membrane protrusions around migrating leukocytes upon RhoA depletion, whereas the F-actin rings were significantly decreased. Suggesting that the basolateral F-actin ring and not the apical protrusions in the endothelial pore contribute to vascular leakage prevention during TEM. Interestingly, in drosophila, similar actomyosin networks have been found to rapidly close multicellular wounds by actomyosin contraction^55^. Studies that investigated the mechanisms by which ECs repair gaps in the endothelial monolayer, show that mechanical induced microwounds in the endothelium are healed by ventral lamellipodia, a mechanism that may also be involved in the closure of leukocyte-induced endothelial pores^56^. Our data show that RhoA-mediated contractile force generation responsible for endothelial pore restriction precedes ventral lamellipodia formation. Moreover, RhoA-mediated pore constriction in ECs seems to be specific for the closure of leukocyte-induced endothelial pores, whereas ventral lamellipodia are also observed in maintenance of basal junctional integrity^57^. On the basis of electron microscopy studies, it has been suggested that the intimate contact between neutrophils and ECs during the entire transendothelial migration process limits leakage of plasma proteins. Moreover, several studies showed that ECs reseal the endothelial barrier before or in conjunction with neutrophils penetrating the basal lamina^15^,^58^. In agreement with these ultrastructural studies we found that endothelial pores closed before or in conjunction with neutrophils that fully breeched the endothelial lining. In the context of EC barrier maintenance it is well conceivable that endothelial pore confinement and closure directly prevents vascular leakage during leukocyte diapedesis whereas ventral lamellipodia restore junctional homeostasis after leukocyte crossing. Endothelial LSP1 has been implicated in a role for ‘dome' formation and controlling permeability during TEM^58^ and has been found to be activated downstream from ICAM-1 clustering^59^. Altogether, this may open up the possibility that ICAM-1 clustering activates RhoA through LSP1. However, future experiments should show if this signalling axis indeed is operational during TEM.

In conclusion, we have discovered that local RhoA-mediated F-actin rings in the endothelial lining prevent vascular leakage during leukocyte diapedesis. Elucidating the molecular and cellular mechanisms of barrier maintenance during leukocyte diapedesis may have implications for the development of new therapies to restore normal homeostatic junctional remodelling to counteract vascular dysfunction during chronic inflammation.

## Methods

### DNA and RNA constructs

The DORA RhoA and DORA RhoA mutant PKN biosensors were a very kind gift of Yi Wu (University of Connecticut Health centre, Farmington, USA). Briefly, circular permutated PKN effector of RhoA coupled to dimeric circular permutated Venus is linked via a ribosomal protein-based linker (L9H) with dimeric Cerulean3 (Cer3) coupled to RhoA. The DORA RhoA sequence within a pTriEx-HisMyc backbone is cpPKN(S69-H97-GSG-S14-R68)-KpnI-GS-dcpVen-L9Hx3-BamHI-dCer3(G229)-NheI-RhoA-WT-HindIII. The DORA RhoA mutant PKN sequence within a pTriEx-HisMyc backbone is cpPKN (S69-H97-GSG-S14-R68, L59Q)-KpnI-GS-dcpVen-L9Hx3-BamHI-dCer3(G229)-NheI-RhoA-WT-HindIII. The Leucine (L) on position 59 in the PKN domain of the RhoA control biosensor is substituted for a glutamine (Q). The H1R, p63-RFP and mRFP-RhoGDI-α (pcDNA 3.1) were a kind gift of Joachim Goedhart (Swammerdam Institute for Life Sciences, University of Amsterdam, Amsterdam, the Netherlands). pLenti-Lifeact-mCherry, pLenti-Lifeact-GFP, were a kind gift of Stephan Huveneers (Sanquin, Amsterdam, the Netherlands). shRNA in pLKO.1 targeting VE-cadherin (12) B6 (TRCN 54090), GEF-H1 (TRCN 3174), GEF-H1 (TRCN 3175), p115RhoGEF (TRCN 33567), and Ect2 (TRCN 47686) were purchased from Sigma Aldrich mission library. siRNA targeting RhoA (sc-29471) (working concentration 50 nM), ICAM-1 sc-29354 (50 nM), PECAM-1 sc-29445 (50 nM), LARG sc-41800 (50 nM), Rock-1 (sc-29473) (50 nM), Rock-2b (sc-29474) (50 nM),and scrambled non-silencing siRNA were purchased from (Santa Cruz Biotechnology, Santa Cruz, CA).

### Antibodies

Rabbit antibody against GEF-H1 (55B6) (Cat #4076) (1:1000 for WB), phosphor-Myosin light-chain Thr18/Ser19 (Cat #3674) (1:100 for IF), p115RhoGEF (D25D2) (Cat #3669) (1:1,000 for WB), RhoA (67B9) (Cat #2117X) (1:1,000 for WB) and CD31 (PECAM-1) (89C2) (Cat #3528) (1:1000 for WB) were purchased from Cell Signaling (BIOKE). Polyclonal rabbit antibody against Ect2 (Cat# 07-1364) (1:1,000 for WB) was purchased from Millipore. Polyclonal goat antibody against LARG (Cat#AF4737) (1:1,000 for WB) was purchased from R&D systems. Alexa Fluor 405 Phalloidin (1:100 for IF) was purchased from Promokine (Cat# PK-PF405-7-01). Polyclonal goat antibody against VE-cadherin (C-19) (Cat# SC-6458) (1:1,000 for WB), Rock-2 (C-20) sc-1851 (1:1,000 for WB), Rock-1 (H-85) sc-5560 (1:1,000 for WB) were purchased from Santa Cruz (Bio-Connect). Polyclonal rabbit antibody against ICAM-1 (Cat #SC-7891) (1:1,000 for WB) was purchased from Santa Cruz Biotechnology. Monoclonal mouse antibody against Filamin A (Cat #MCA464S) (1:1,000 for WB) was purchased from Serotec. Monoclonal mouse Alexa Fluor 647 VE-cadherin (55-7H1) ( Cat# 560411) (1:100 for IF) and Alexa Fluor 488 PECAM-1 (Cat# 555445) (1:100 for IF) were purchased from Becton Dickinson. Monoclonal mouse antibody against Actin (AC-40) (Cat# A3853) (1:1,000 for WB) was purchased from Sigma. The Alexa Fluor 405 goat anti-rabbit IgG (Cat# A31556) (1:100 for IF), Alexa Fluor 647 chicken anti-goat IgG (Cat# A21469) (1:100 for IF), Alexa Fluor 488 chicken anti-rabbit IgG (Cat# A21441) (1:100 for IF) and Texas red 568 Phalloidin (Cat #T7471) (1:100 for IF) were purchased from Invitrogen. Secondary HRP-conjugated goat anti-mouse, swine anti-rabbit antibodies (1:3,000 for WB) were purchased from Dako (Heverlee, Belgium). Hoechst 33342 (H-1399) (1:50 for IF) was purchased from Molecular probes (Life Technologies). All antibodies were used according to manufacturer's protocol.

### Cell cultures and treatments

Pooled human umbilical vein ECs (HUVECs) purchased from Lonza (P938, Cat # C2519A), were cultured on fibronectin (FN)-coated dishes in EGM-2 medium, supplemented with singlequots (Lonza, Verviers, Belgium) HUVECs were cultured until passage 9. HEK-293T were maintained in DMEM (Invitrogen, Breda, The Netherlands) containing 10% (v/v) heat-inactivated fetal calf serum (Invitrogen, Breda, The Netherlands), 300 mg ml^−1^ L-glutamine, 100 U ml^−1^ penicillin and streptomycin and 1 × sodium pyruvate (Invitrogen, Breda, The Netherlands). HeLa cells (American Tissue Culture Collection: Manassas, VA, USA) were cultured using DMEM supplied with Glutamax, 10% fetal bovine serum, Penicillin (100 U ml^−1^) and Streptomycin (100 μg ml^−1^). Cells were cultured at 37 °C and 5% CO_2_. HUVECs were treated with 1 U ml^−1^ thrombin (Sigma-Aldrich, St Louis, USA) for periods as indicated, pretreated with 10 ng/ml recombinant TNF-α (PeproTech, Rocky Hill, NJ) 24 h before each leukocyte TEM experiment, For Rho inhibition cells were preincubated with cell-permeable Rho inhibitor I (C3) (Cytoskeleton, Cat# CT04) for 3 h. Cells were transfected with the expression vectors according to the manufacturer's protocol with Trans IT-LT1 (Myrus, Madison, WI, USA). Lentiviral constructs were packaged into lentivirus in Human embryonic kidney (HEK)-293T cells by means of third generation lentiviral packaging plasmids (Dull *et al.*, 1998; Hope *et al.* 1990). Lentivirus containing supernatant was collected on day 2 and 3 after transfection. Lentivirus was concentrated by Lenti-X concentrator (Clontech, Cat# 631232). Transduced target cells were used for assays after 72 h. Cells were transfected with siRNA according to manufacturer's protocol using INTERFERin (Polyplus). HeLa cell were transfected with Lipofectamine and imaged the next day. HeLa cells were treated with 100 μM histamine (Sigma-Aldrich, St Louis, USA) and 10 μM Pyrilamine (mepyramine; Sigma-Aldrich, St Louis, USA) for periods as indicated.

### Neutrophil and monocyte isolation

Polymorphonuclear neutrophils and monocytes were isolated from whole-blood derived from healthy donors who signed an informed consent under the rules and legislation in place within the Netherlands and maintained by the Sanquin Medical Ethical Committee. The rules and legislations are based on the Declaration of Helsinki (informed consent for participation of human subjects in medical and scientific research) and guidelines for Good Clinical Practice. Whole blood was diluted (1:1) with 5% (v/v) TNC in PBS. Diluted whole blood was pipetted carefully on 12.5 ml Percoll (room temperature) 1.076 g ml^−1^. Tubes were centrifuged (Rotanta 96R) at 2,000 r.p.m., slow start, low brake for 20 min. Ring fraction containing lymphocytes and monocytes was collected and further processed as indicated below*. After erythrocyte lysis in an ice-cold isotonic lysis buffer (155 mM NH_4_CL, 10 mM KHCO_3_, 0.1 mM EDTA, pH7.4 in Milli-Q(Millipore), neutrophils were centrifuged at 1,500 r.p.m. for five minutes at 4 °C, incubated once with lysis buffer for 5 min on ice, centrifuged again at 1,500 r.p.m. for 5 min at 4 °C, washed once with PBS, centrifuged again at 1,500 r.p.m. for 5 min at 4 °C and resuspended in HEPES medium (20 mM HEPES, 132 mM NaCl, 6 mM KCL, 1 mM CaCL_2_, 1 mM MgSO_4_, 1.2 mM K_2_HPO_4_, 5 mM glucose (all from Sigma-Aldrich), and 0.4 % (w/v) human serum albumin (Sanquin Reagents), pH7.4) and kept at room temperature for not longer than 4 h until use. *Ring fraction was washed three times with MACS buffer (0.5% (v/v) bovine serum albumin (BSA) in PBS, 2 mM EDTA in PBS). Ring fraction was centrifuged at 1,700 r.p.m. for 7 min at 4 °C and low break, and resuspended in 100 μl MACS buffer and incubated with 5 μl CD14 microbeads (Miltenyi biotec, # 130-050-201) for 30 min at 4 °C and subsequently washed with 5 ml MACS buffer, centrifuged and resuspended in 1 ml MACS buffer. LS columns (Miltenyi biotec, # 130-042-401) were placed in QuadroMACS separator (Miltenyi biotec, # 130-090-976) and cells were subsequently washed three times with 1 ml MACS buffer. Column was removed from the QuadroMACS separator and monocytes were collected in a collection tube. Neutrophil and monocyte counts were determined by cell counter (Casey).

### FITC–dextran permeability assay

ECs (*n*=200,000) were cultured in FN-treated 24-well cell culture inserts (Corning FluoroBlok, Falcon, 3.0-μm pore size # 351151) and treated with TNF-α overnight. 30 μg FITC–dextran (70 kDa; Sigma) in HEPES medium (20 mM HEPES, 132 mM NaCl, 6 mM KCL, 1 mM CaCL_2_, 1 mM MgSO_4_, 1.2 mM K_2_HPO_4_, 5 mM glucose (all from Sigma-Aldrich), and 0.4 % (w/v) human serum albumin (Sanquin Reagents), pH7.4) was added to the upper and 0.1 nM C5a (Sigma C-5788) in HEPES medium was added to the lower compartment. FITC–dextran and calcein red–orange (Molecular probes C34851) labelled neutrophil (200,000 cells) extravasation was monitored simultaneously for a period of 60 min with an interval of 1 min by an Infinite F200 pro plate reader (TECAN) at 37 °C. EX BP 485/9 and EM BP 535/20 was used to measure FITC–dextran kinetics. EX BP 535/9 and EM BP 595/20 was used to measure neutrophil (calcein red–orange) transmigration kinetics.

### Neutrophil and monocyte TEM under physiological flow

HUVECs were cultured to 70% confluence in FN-coated 6-well plate, and transfected with different expression vectors (for example, DORA RhoA biosensor) according to the manufacturer's protocol with Trans IT-LT1 (Myrus, Madison, WI, USA) for 24 h or transduced with expression vectors in lentivirus (for example, pLenti-Lifeact-mCherry) for 72 h. HUVECs were cultured in a FN-coated ibidi μ-slide VI^0.4^ (ibidi, Munich, Germany) the day before the experiment was executed and stimulated overnight with TNFα (10 ng ml^−1^). Freshly isolated neutrophils and monocytes were resuspended at 1 × 10^6^ cells per ml in HEPES medium and were incubated for 30 min at 37 °C. Cultured HUVECs in ibidi flow chambers were connected to a perfusion system and exposed to 0.5 ml min^−1^ HEPES shear flow for 10 min (0.8 dyne per cm^2^). Actual levels of injected neutrophils ranged between 0.5 and 2 × 10^6^ dependent on the donor and activity of the neutrophils. Actual levels of monocytes ranged between 1.5 and 2.2 × 10^6^. Neutrophils or monocytes were subsequently injected into the perfusion system and real-time leukocyte–endothelial interactions were recorded for 20 min by a Zeiss Observer Z1 microscope using a 40 × numerical aperture (NA) 1.3 oil immersion objective or samples were fixed for immunofluorescent staining and subsequent analysis. All live imaging was performed at 37 °C in the presence of 5% CO_2_. Transmigrated neutrophils were distinguished from those adhering to the apical surface of the endothelium by their transition from bright to phase-dark morphology. Percentage adherent or transmigrated neutrophils were manually quantified using the ImageJ plug-in Cell Counter (type 1, adherent cells, type 2, transmigrated cells).

### Confocal laser scanning microscopy

Cells were cultured in FN-coated ibidi μ-slide VI^0.4^ (ibidi, Germany) and transfected or stimulated as indicated. After treatment, cells were washed with cold PBS, containing 1 mM CaCl_2_ and 0.5 mM MgCl_2_, and fixed in 4% (v/v) formaldehyde for 10 min. After fixation, cells were permeabilized in PBS supplemented with 0.5% (v/v) Triton X-100 for 10 min followed by a blocking step in PBS supplemented with 2.5% (w/v) BSA. Cells were incubated with primary and secondary antibodies and after each step washed with PBS. Z-stack image acquisition was performed on a confocal laser scanning microscope (LSM510/Meta; Carl Zeiss Micro-Imaging) using a voxel size of 0.06 × 0.06 × 0.48 μm and a 63 × NA 1.4 oil immersion objective. Following acquisition, the sequences of Z-stack images were analysed off-line using Imaris, which renders the optical sections into 3D models enabling analysis of leukocyte–endothelial interaction dynamics.

### Quantification endothelial pore structures

Real-time endothelial pore dynamics in HUVECs expressing Lifeact-GFP was analysed by ImageJ. Three parameters were scored; the area of the neutrophil cell body (luminal site), the area of the pore, and the area of the neutrophil cell body under the endothelium (abluminal site). The area (μm^2^) of each parameter was measured manually by drawing a region of interest (ROI) resembling the parameter (for example, pore size) and measured in ImageJ using analyse measure. Endothelial pore sizes of fixed HUVECs were measured by drawing a ROI resembling various parameters (width, length, height, circularity and pore size). Length of the endothelial pore was defined as the total distance of the pore parallel to the junction and width was defined as the total distance perpendicular to the junction. Circularity of the pore is defined by the length over width ratio. Pore size was defined as the total surface area of endothelial pore. Total F-actin in the pore (sum intensity Z-slices) was divided in two equal ROI. Distribution asymmetry was defined by ROI (1)-BG over ROI (2)-BG ratio. Leukocytes migrating through the EC body not interrupting junctional VE-cadherin were scored as transcellular migration, whereas leukocyte migrating between ECs interrupting junctional VE-cadherin were scored as paracellular migration.

### Characterization of DORA RhoA biosensor

The design of the DORA RhoA sensor is based on the published RhoA biosensor^20^. Importantly, the GTPase is placed at the C terminus of the construct enabling the DORA RhoA biosensor to localize at the plasma membrane similar to endogenous RhoA (Fig. 2a). FRET efficiency of the ON-state of the GTPase is improved through modelling of the fluorescent protein dimers and the GTPase-effector domain complexes. Repeats of stable α-helix from ribosomal protein L9, rather than an unstructured linker, is inserted between the fluorescent proteins to disrupt dimerization and diminish FRET efficiency in the inactive state (Fig. 2a). In ECs, the DORA RhoA sensor accurately reported thrombin induced RhoA activation, showing a fast and transient RhoA activation (Fig. 2b, Supplementary Fig. 1f, Supplementary Movie 7). Quantification of 10 independent experiments showed reproducible RhoA activation patterns on thrombin treatment (Supplementary Fig. 1f). As a control, DORA RhoA mutant PKN was generated to report misalignment of Cer3 and Venus image before and after image registration, motion artefacts or pH changes affecting the sensors fluorescent proteins. The substitution of glutamine for the leucine at position 59 in PKN prevents the binding of PKN to activated RhoA, in the DORA RhoA mutant PKN^21^. The DORA RhoA mutant PKN showed no change in Venus/Cer3 ratio after thrombin stimulation (Supplementary Fig. 2a; Supplementary Movie 8). Quantification of 10 independent experiments showed no change in Venus/Cer3 ratio after thrombin addition in ECs that expressed the DORA RhoA mutant PKN biosensor (Supplementary Fig. 2a). In addition, Hela cells that overexpressed the histamine receptor, histamine stimulation induced a fast and sustained RhoA activation (Supplementary Fig. 2b; Supplementary Movie 9). Subsequent addition of Pyrilamine reduced RhoA activity to baseline levels, showing that the sensor dynamically reports RhoA activation and inactivation^60^. The DORA RhoA mutant PKN showed no change in Venus/Cer3 ratio after histamine stimulation (Supplementary Fig. 2c; Supplementary Movie 10). To show that the sensor is cycling between an active and inactive, Rho-GDI-bound form, we co-expressed Rho-GDI and found a reduced FRET ratio, illustrative for binding to Rho-GDI (Supplementary Fig. 3a). In conclusion, the DORA RhoA biosensor accurately reports RhoA dynamics in ECs. Important to note, Venus and Cer3 emission were simultaneously recorded utilizing a double-camera system, since sequential image acquisition results in motion artefacts induced by migrating leukocytes displacing fluorescent signals in the ECs.

### Characterization of DORA FRET RhoA-sensor

The intrinsic single-chain nature of the DORA RhoA biosensor rules out the possibility of unequal Cer3 and Venus localization within the EC. Therefore, DORA RhoA sensor translocation always results in a paired translocation of Cer3 and Venus emission. In case the biosensor does translocate (for example, in response to EC shape alterations induced by migrating neutrophils lowering the local Venus and Cer3 emission dramatically) sequential imaging may result in erroneous measurements. The delay in the acquired Cer3 and Venus emission image of around 400–800 ms may therefore contain an intrinsic local CFP-YFP pixel shift that cannot be solved by registration software. Therefore, quality of the obtained Venus/Cer3 Ratio acquired by sequential image acquisition is dependent on the transmigration speed of the neutrophil. To control for erroneous measurements during sequential image acquisition one could change the acquisition order of sequential image acquisition from Cer3-Venus to Venus-Cer3, the DORA RhoA activity status will change from high- to a low-activity pattern. If the activity pattern alters, then it indicates that an intrinsic local CFP-YFP pixel shift is present in the sequentially acquired Cer3 and Venus dataset. To circumvent motion artefacts a dual-camera set-up was used to acquire Cer3 and Venus emission images simultaneously. In our set-up, photobleaching-rates of Cer3 and Venus are more or less equivalent. Photobleaching (average±s.d.) was determined by the reduction in fluorescence (1-F/F0) in living cells after 30 min illumination with HXP V at 20%, 800 ms illumination with an interval of 5 s. dCer3 photo bleaching was 16.3%±7.6%) and dcpVenus bleaching was 13.3%±7.9%). All ratiometric images and normalized intensity graphs were not corrected for photo bleaching, since photobleaching kinetics may change when FRET changes. Cer3 bleed through into the Venus channel is 57%. All normalized intensity graphs were corrected for bleed through using the equation (YFP-BG)-(0.57*(CFP-BG).

### DORA FRET RhoA-sensor analysis

We use a Zeiss Observer Z1 microscope with 40 × NA 1.3 oil immersion objective, a HXP 120-V excitation light source, a Chroma 510 DCSP dichroic splitter, and two Hamamatsu ORCA-R2 digital CCD cameras (2 × 2 binning) for simultaneous monitoring of Cer3 and Venus emissions. Image acquisition was performed using Zeiss Zen 2011 microscope software. The lowest achievable HXP excitation power, through a FRET filter cube (Exciter ET 436/20 × , and 455 DCLP dichroic mirror (Chroma), the emission filtre is removed) was used to excite the Cer3 donor. The emission is directed to the left side port by a 100% mirror, to an attached dual-camera adaptor (Zeiss) controlling a 510 DCSP dichroic mirror. Emission wavelengths between 455 and 510 nm are directed to an emission filter (ET 480/40, Chroma) and then captured by the ‘straight' Hamamatsu ORCA-R2 camera resulting in Cer3 image acquisition. The Emission wavelength 510 nm and higher are directed to a six positions LEP filter wheel (Ludl Electronic Products) placed in front of the second ‘rear' Hamamatsu ORCA-R2 camera. Position 1 in the emission filter wheel is equipped with an ET 540/40 m used for venus image acquisition. Position 2 is left empty to allow mCherry image acquisition (EX BP 572/25, BS FT 590, EM BP 629/62, Zeiss). The LEP filter wheel is controlled by the MAC 6000 controller system (Ludl Electronic Products). Exposure time of DIC image was set to 76 ms and exposure time of simultaneous CFP and YFP acquisition was set to ±800 ms, images were subsequently recorded every 5 s for periods as indicated. Offline ratio analysis between Cer3 and Venus images was done utilizing the MBF ImageJ collection (Tony Collins). Individual images in the raw Cer3 and Venus image stacks were background (BG)-corrected using the plug-in ‘ROI, BG subtraction from ROI'. An ROI in the image were no cells were present was selected as the background. Next, the Cer3 and Venus stacks were aligned using the registration plug-in ‘Registration, MultiStackReg'. A smooth filtre was applied to both image stacks to improve image quality by reducing the noise. The smooth filtre (mean filtre) was applied over the entire image and replaces each pixel with the average of its 3 × 3 neighbourhood. Next, both image stacks were converted to a 32-bit image format, required for subsequent masking. A user defined threshold was applied exclusively to the Venus image stack, converting the background pixels to ‘not a number' (NaN). It allows elimination of artifacts in ratio image stemming from the background noise. Finally the Venus/Cer3 ratio was calculated using the plug-in ‘Ratio Plus', and a custom lookup table was applied to generate a colour-coded image illustrating the high ‘red' and low ‘blue' activities. Note that some of the plug-ins, namely MultiStackReg, and Ratio Plus are not included in the basic MBF ImageJ collection and should be downloaded from the plug-in page in the ImageJ website ( http://rsb.info.nih.gov/ij/plugins/index.html). Normalized intensity graphs; the intensity of an manually selected ROI of interest and the BG of the raw Cer3 and Venus image stacks were measured using the plug-in ROI, Multi Measure in ImageJ. The raw Cer3 and Venus intensities were BG subtracted using equation Cer3=(Cer3 raw−BG), subsequently Venus only was corrected for bleed through using the equation VenusC=(Venus)−(0.5748*(Cer3). Normalization of the individual intensity traces was done by dividing the data by the average over acquisition 3 to 13. The Venus/Cer3 ratio was calculated from the normalized data using Excel.

### Confocal intravital microscopy of mouse cremaster muscles

To investigate local myosin light-chain phosphorylation during leukocyte diapedesis, whole-mounted cremaster muscles of mice expressing endogenously labelled GFP-leukocytes (*Lys-EGFP-ki, CX3CR1-EGFP-ki*, Tie-2Cre) or C57BL6 mice were fixed, immunostained and analysed by confocal microscopy. Inflammation in mouse cremaster muscles was induced by intrascrotal injection (i.s.) of IL-1β/TNF-α (50 ng IL-1β, 300 ng TNF-α in 400μl saline) or saline (400 μl) for 2 h. EC junctions were labelled through co-administration of Alexa Fluor-555 or 647-labelled anti-PECAM-1 mAb (clone 390; 3 μg i.s.). Anti-PECAM mAb clone 390 was conjugated to Alexa Fluor-555 using a commercially available kit (Invitrogen, Paisley, UK). The cremasters were surgically exteriorized and TEM was analysed by intravital microscopy. Straight post-capillary venules of 20–40 μm in diameter were selected for analysis of leukocyte-vessel wall interactions. Z-stacks of images were captured by confocal microscopy as described^23^. The tissues were fixed with 4% paraformaldehyde for 30 min at a time point with several paracellular pores visible in the PECAM-1 labelling. Subsequently, tissues were permeabilized with 0.5% Triton and blocked with 10% BSA and 10 μg μl^−1^ FC block for 2 h. Tissues were incubated with anti-phospho Myosin light-chain Thr18/Ser19 (Cat #3674) purchased from Cell signaling (BIOKE) antibody for 2 days and a further 2 days with the secondary antibody or IgG isotype control (1:100). Transmigration induced permeability was examined by looking at fluorescent TRITC–dextran leakage into the cremaster of C57BL/6 WT or LysM–GFP mice during IL-1β and TNFα stimulated neutrophil recruitment. Four different groups were defined; unstimulated, Rho inhibitor I (C3) alone, IL-1β/TNF-α treated, IL-1β/TNF-α treated+C3. IL-1β (50 ng), TNF-α (300 ng), Rho-inhibitor (1 μg) and anti-PECAM-1 labelling antibody (4 μg) were given intrascrotally at *T*=0 h. A second dose of C3 (1 μg) was given intrascrotally and TRITC–dextran (40 kDa, 200 μl/40 μM) was injected intravenously at *T*=2 h and allowed to circulate until *T*=4 h. At *T*=4 h animals were sacrificed and tissues were subjected to vascular wash out by perfusion of 10 ml PBS via the left ventricle. Tissues were examined by confocal microscopy (Leica SP5, 20 × objective) and the level of TRITC–dextran fluorescence by region of interest in the surrounding tissue was quantified using the LAS-AF Lite software. Neutrophil extravasation per 5 × 10^4^ μm^2^ tissue area was quantified by endogenous GFP fluorescence in the neutrophils of LysM–GFP mice, or by fixation and labelling of neutrophils with anti-MRP14-alexa647 in WT mice after analysis of TRITC–dextran leakage in unfixed tissues. All *in vivo* experiments followed UK legislation for the protection of animals and were approved by the Ethical Review Process of Queen Mary, University of London.

### F-actin visualization in retinal vasculature of Lifeact-GFP mice

Lifeact-EGFP transgenic mice have been previously described^26^. All experiments with mice were performed in accordance to German guidelines and regulations. Mice were analysed at p7 and gender was randomly picked. The protocol was approved by the Committee on the Ethics of Animal Experiments of the Ludwig-Maximilians University Munich.

### Whole-retina immunohistochemistry

Dissection and labelling of retinas was performed as previously described^27^. Briefly, retinas were fixed for 2 h on ice in 4% paraformaldehyde, incubated in 1% BSA and 0.3% Triton X-100, washed two times in Pblec (1% Triton X-100, 1 mM CaCl2, 1 mM MgCl2, and 1 mM MnCl2 PBS (pH 6.8)), and incubated overnight with isolectin-B4 (1:50) and antibodies diluted in Pblec. Images were acquired and processed using a Leica TCS SP5 II microscope, LAS Montage Imaging software (Leica) and the IMARIS Digital Imaging software (Biplane). Biotinylated *Griffonia simplicifolia* lectin I (IB4), (B-1205) was purchased from Vector Laboratories.

### Western blotting

Cells were washed twice with PBS, and lysed with 95 °C SDS-sample buffer containing 4% β-mecapto-ethanol. Samples were boiled at 95 °C for 4 min to denature proteins. Proteins were separated on 4–12% NuPAGE Novex Bis-Tris gels (Invitrogen), transferred to Immobilon-PVDF transfer membranes (Millipore Corp., Billerica, MA) and subsequently blocked with 2.5% (w/v) BSA in Tris-buffered saline with Tween 20 for 60 min. The immunoblots were analysed using primary antibodies incubated overnight at 4 °C and secondary antibodies linked to horseradish peroxidase (HRP) (GE Healthcare, UK), after each step immunoblots were washed 6 × with Tris-buffered saline with Tween 20. Signals were visualized by enhanced chemiluminescence and light sensitive films (GE Healthcare, UK). The full blots of all immunoblots presented in the study are shown in Supplementary Fig. 8.

### Pull-out assay and immunoprecipitation

Volume of 1.2 mg ml^−1^ dynabeads goat-x-ms IgG (Dynal, Invitrogen) per condition were washed ones with 1 ml buffer 1 containing PBS+2 mM EDTA and 0.1% BSA (Millipore) using a magnetic holder. Dynabeads were coated with 1.6 μg α-ICAM-1 CD54 (BBIG-I1/IIC81, R&D systems (Cat #BBA9) antibodies per condition and incubated head-over-head at 4 °C for 45 min. The beads were washed twice using buffer 1 and resuspended in PBS containing 0.5 MgCl_2_ and 1 mM CaCl_2_. Overnight TNF-α treated (10 ng ml^−1^) HUVEC (2–5 million cells) were pretreated with 1 μM Ionomycin (Invitrogen, I-24222) for 10 min. 1.2 mg ml^−1^ dynabeads per condition were used to cluster ICAM-1 for 10–30 min. Cells were washed once on ice using PBS containing 0.5 MgCl_2_ and 1 mM CaCl_2_. Next, cells were lysed in 1 ml cold pH7.4 RIPA buffer containing 50 mM Tris, 100 mM NaCl, 10 mM MgCl_2_, 1% NP40, 10% glycerol, 0.1% SDS, 1% DOC (Sigma-Aldrich), DNAse inhibitor and protease phosphatase inhibitor cocktail for 5 min. Cells were scraped together and lysates were transferred to a new tube. Then, ICAM-1 coated dynabeads were added to non-clustered-control cells. 50 μl whole-cell lysate was taken from all conditions. Beads and cell-lysates were subsequently incubated head-over-head for 1–2 h at 4 °C. Next cells were washed twice with Ripa buffer and three times with NP-40 lysis buffer. Beads were resuspended in 30 μl 2 × SDS-sample buffer and assessed by western blotting.

### Electric cell–substrate impedance sensing under flow

Endothelial monolayer integrity during leukocyte diapedesis was determined by measuring the electrical resistance using Electric Cell–substrate Impedance Sensing (ECIS). Flow chamber electrode arrays (8F10E; Applied Biophysics, Troy, NY) were pretreated with 10 mM L-cysteine (Sigma-Aldrich) for 15 min at 37 °C, subsequently washed twice with 0.9% NaCl and coated with FN (Sanquin) in 0.9% NaCl for 1 h at 37 °C. Cells were seeded at 300,000 cells per slide (2.5 cm^2^) and grown to confluence. Continuous resistance measurements were performed at 37 °C at 5% CO_2_ with the ECIS ZΘ (Theta) system controller (Applied Biophysics). After forming a stable monolayer, HUVECs were subjected to flow (0.8 dynes per cm^2^) for periods as indicated.

### Statistics

Endothelial pore analyses between leukocyte subtypes was tested using a one-way analysis of variance assuming no matching or pairing, comparing the mean of each column with the mean of every other column, that were corrected for multiple comparisons by Tukey's multiple comparisons test, with a single pooled variance. Statistical comparison between experimental groups was performed by the Student's *T*-test. A two-tailed *P* value of <0.05 was considered significant. Correlation analysis of dextran and neutrophil extravasation kinetics was performed by Pearson's correlation. Unless otherwise stated, a representative experiment out of at least three independent experiments is shown.

## Additional information

**How to cite this article:** Heemskerk, N. *et al.* F-actin-rich contractile endothelial pores prevent vascular leakage during leukocyte diapedesis through local RhoA signalling. *Nat. Commun.* 7:10493 doi: 10.1038/ncomms10493 (2016).

## Supplementary Material

Supplementary Figures Supplementary Figures 1-8

Supplementary Movie 1 Endothelial RhoA is locally and transiently activated during neutrophil extravasation not during adhesion. Time-lapse recordings of the DORA RhoA biosensor showing spatiotemporal RhoA activation during neutrophil TEM under physiological flow conditions (0.8dyne per cm2). Left panels from top to bottom show, Venus/Cer3 ratio images of the DORA RhoA biosensor, Merge and DIC, respectively. Right panels show zoom of the ROI shown in left panels. Calibration bar shows RhoA activation (Red) relative to basal RhoA activity (Blue). Images were simultaneously acquired by time-lapse wide-field microscopy (Zeiss Observer Z1) using a 40x 1.3 NA oil immersion objective. Frames were taken every 4 s for 6 minutes.

Supplementary Movie 2 Spatiotemporal activation of DORA RhoA mutant PKN biosensor during neutrophil TEM. Time-lapse recordings of the DORA RhoA mutant PKN biosensor showing no change in spatiotemporal RhoA activation during neutrophil extravasation under physiological flow conditions (0.8dyne per cm2). Left panels from top to bottom show, Venus/Cer3 ratio images of the DORA RhoA mutant PKN biosensor, Merge and DIC, respectively. Right panels show zoom of the ROI shown in left panels. Calibration bar shows RhoA activation (Red) relative to basal RhoA activity (Blue). Images were simultaneously acquired by time-lapse wide-field microscopy (Zeiss Observer Z1) using a 40x 1.3 NA oil immersion objective. Frames were taken every 5 s for 9 minutes.

Supplementary Movie 3 F-actin rich endothelial pore structure around paracellular migrating monocyte. 3D representation of confocal Z-stack showing Lifeact-GFP (green) and Lifeact-mCherry (red) positive endothelial membrane structures that together form a paracellular endothelial pore. The endothelial pore has filopodia-like protrusions at the apical site and a cortical actin-ring at the basolateral site. VE-cadherin (white) distribution at the junction is discontinuous at the site where the monocyte breeches the EC junction. DAPI staining shows extravasating monocyte (blue). Z-stack images were acquired by confocal laser scanning microscopy (LSM510/Meta; Carl Zeiss Micro-Imaging) using a voxel size of 0.06x0.06x0.48 μm and a 63x 1.4 NA oil immersion objective. Following acquisition, the sequences of Z-stack images were analyzed off-line using Imaris which renders the optical sections into 3D models enabling analysis of leukocyte-endothelial interactions.

Supplementary Movie 4 F-actin rich endothelial pore structure around paracellular migrating monocyte lacking F-actin rich apical protrusions. 3D representation of confocal Z-stack showing Lifeact-GFP (green) endothelial pore around paracellular migrating monocyte. VE-cadherin (white) distribution is discontinuous at the site where the monocyte breeches the EC junction. DAPI staining shows extravasating monocyte (blue). Note, adherent monocytes do not induce F-actin structures at the apical site of the endothelium. Z-stack images were acquired by confocal laser scanning microscopy (LSM510/Meta; Carl Zeiss Micro-Imaging) using a voxel size of 0.06x0.06x0.48 μm and a 63x 1.4 NA oil immersion objective. Following acquisition, the sequences of Z-stack images were analyzed off-line using Imaris which renders the optical sections into 3D models enabling analysis of leukocyte-endothelial interactions.

Supplementary Movie 5 F-actin rich endothelial pore structure around transcellular migrating monocytes. 3D representation of confocal Z-stack showing Lifeact-GFP (green) endothelial pores around transcellular migrating monocyte. VE-cadherin (white) distribution at the junction is continuous. DAPI staining shows extravasating monocytes (blue) through the EC cell body. Z-stack images were acquired by confocal laser scanning microscopy (LSM510/Meta; Carl Zeiss Micro-Imaging) using a voxel size of 0.06x0.06x0.48 μm and a 63x 1.4 NA oil immersion objective. Following acquisition, the sequences of Z-stack images were analyzed off-line using Imaris which renders the optical sections into 3D models enabling analysis of leukocyte-endothelial interactions.

Supplementary Movie 6 Endothelial F-actin and VE-cadherin dynamics during neutrophil diapedesis under physiological flow conditions (0.8dyne per cm2). Real-time confocal recordings of transmigrating neutrophils through ECs expressing GFP-tagged Lifeact (green) showed increased F-actin assembly around endothelial pores. Neutrophils tend to breech junctions containing low amount of VE-cadherin (red) molecules. Images were acquired by confocal laser scanning microscopy (LSM510/Meta; Carl Zeiss Micro-Imaging) using a 63x 1.4 NA oil immersion objective. Frames were taken every 14 s for 7 minutes.

Supplementary Movie 7 Spatiotemporal activation of DORA RhoA biosensor upon thrombin treatment in EC. Time-lapse Venus/Cer3 ratio images of DORA RhoA biosensor simultaneously recorded with an epifluorescent microscope showing spatiotemporal RhoA activation after thrombin stimulation (1U per ml) in HUVECs. Calibration bar shows RhoA activation (Red) relative to basal RhoA activity (Blue). Images were simultaneously acquired by time-lapse wide-field microscopy (Zeiss Observer Z1) using a 40x 1.3 NA oil immersion objective. Frames were taken every 0.3 s for 5 minutes.

Supplementary Movie 8 Spatiotemporal activation of DORA RhoA mutant PKN biosensor upon thrombin treatment in EC. Time-lapse Venus/Cer3 ratio images of DORA RhoA mutant PKN biosensor simultaneously recorded with an epifluorescent microscope showing spatiotemporal RhoA activation after thrombin stimulation (1U per ml) in HUVECs. Calibration bar shows RhoA activation (Red) relative to basal RhoA activity (Blue). Left panel shows Venus/Cer3 ratio images and right panel shows CFP emission only. Images were simultaneously acquired by time-lapse wide-field microscopy (Zeiss Observer Z1) using a 40x 1.3 NA oil immersion objective. Frames were taken every 5 s for 15 minutes.

Supplementary Movie 9 Spatiotemporal activation of RhoA upon histamine stimulation in EC. Time-lapse Venus/Cer3 ratio images of DORA RhoA biosensor after histamine and subsequent antagonist pyrilamine stimulation in Hela cells expressing the histamine receptor. Calibration bar shows RhoA activation (Red) relative to basal RhoA activity (Blue). Left panel shows Venus/Cer3 ratio images and right panel shows CFP emission only. Images were simultaneously acquired by time-lapse wide-field microscopy (Zeiss Observer Z1) using a 40x 1.3 NA oil immersion objective. Frames were taken every 5 s for 15 minutes.

Supplementary Movie 10 Spatiotemporal activation of DORA RhoA mutant PKN biosensor upon histamine stimulation in EC. Time-lapse Venus/Cer3 ratio images of DORA RhoA mutant PKN biosensor after histamine and subsequent antagonist pyrilamine stimulation in Hela cells expressing the histamine receptor. Calibration bar shows RhoA activation (Red) relative to basal RhoA activity (Blue). Left panel shows Venus/Cer3 ratio images and right panel shows CFP emission only. Images were simultaneously acquired by time-lapse wide-field microscopy (Zeiss Observer Z1) using a 40x 1.3 NA oil immersion objective. Frames were taken every 5 s for 15 minutes.
