# Supplementary material for: F-actin-rich contractile endothelial pores prevent vascular leakage during leukocyte diapedesis through local RhoA signalling
Source: Nat Commun. 2016 Jan 27;7:10493. doi: 10.1038/ncomms10493 (PMC4737874; doi:10.1038/ncomms10493)
Supplement: Supplementary Figures — 1-8 [file ncomms10493-s1.pdf]

Supplementary Information

Supplemental Figure 1.

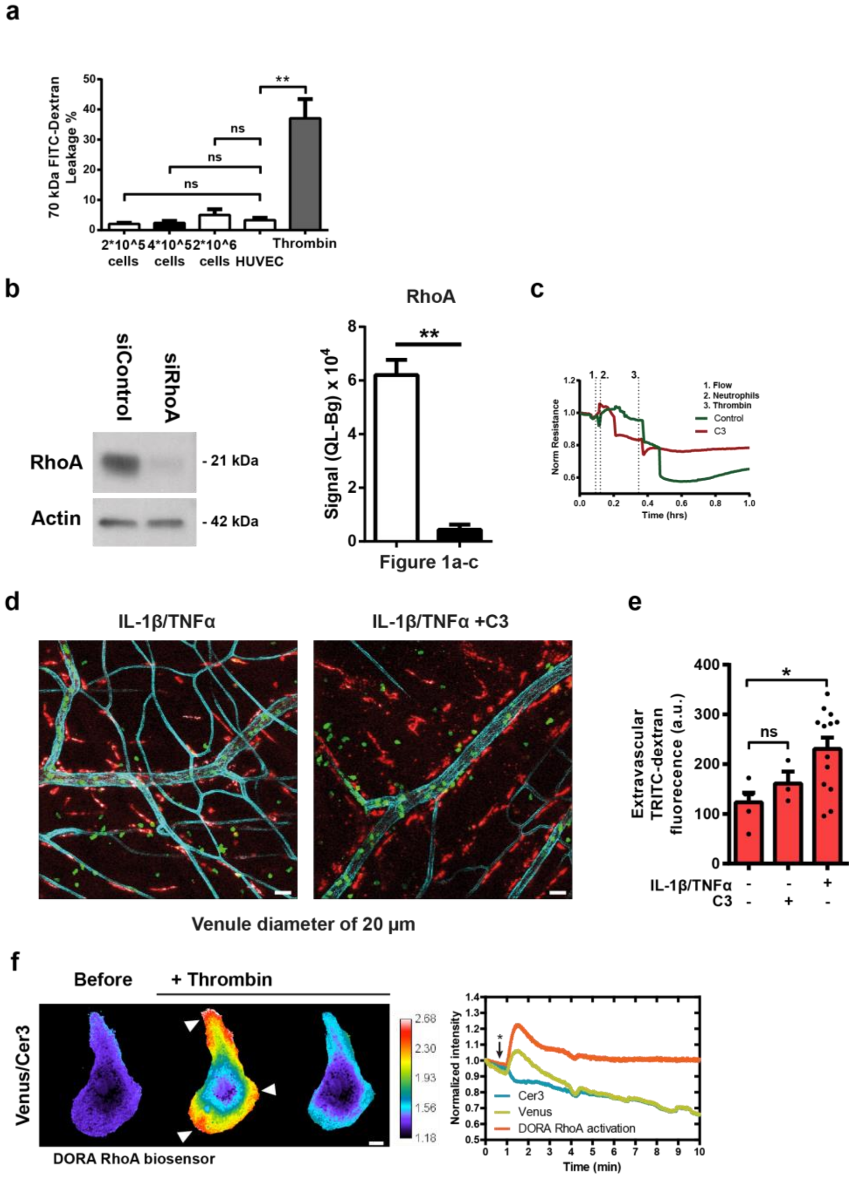

Supplementary Figure 1 Neutrophil transmigration through ECs is associated with minimal FITC-dextran leakage. (**a**) Quantification of FITC-dextran (70 kDa) leakage during neutrophil diapedesis through TNF-α treated HUVECs cultured on 3µm pore permeable filters. Neutrophils transmigrated towards a C5a chemotactic gradient in the lower compartment. (**b**) Endothelial RhoA depletion by siRNA. Immunoblot analysis of protein extracts prepared from HUVEC treated with TNF-α overnight. Extracts were prepared 72 hours after transfection with control and RhoA siRNA duplexes. Extracts were probed with antibodies directed against RhoA and actin. Quantification of RhoA depletion, presented as 'quantum level' (sum of grayness of each pixel in blot) minus background (QL-Bg), normalized to that of actin. (**c**) Normalized resistance of endothelial monolayer during leukocyte diapedesis through HUVECs treated with or without C3 inhibitor measured with ECIS in real-time under flow conditions. Green and red line indicates untreated and C3-pretreated ECs, respectively. Lines represent average of eight wells. Numbers: 1: Onset of flow (0.8 dyne per cm^2); 2: addition of neutrophils (approximately $1*10^6$ per mL); 3: addition of thrombin (1U per mL). (**d**) Confocal intravital microscopy of 20 µm diameter cremasteric venules in LysM-GFP mice (green neutrophils) immunostained *in vivo* for EC junctions by intrascrotal injections of fluorescent-labeled PECAM-1 (blue) and stimulated for four hours with IL-1β and TNF-α only, or with Rho-inhibitor (C3). A second dose of Rho inhibitor was given intrascrotally and TRITC-dextran (40 kDa) was injected intravenously at T = 2 hours and allowed to circulate until T = 4 hours. Scale bar 100µm. (**e**) Quantification of TRITC-dextran extravasation in animals left unstimulated (control), stimulated with C3 alone or IL-1β/TNFα. (**f**) Characterization of DORA RhoA biosensor and DORA RhoA mutant PKN biosensor. Time-lapse Venus/Cer3 ratio images of DORA RhoA biosensor after thrombin treatment in HUVECs. Filled arrows indicate RhoA activation. Scale bar, 10µm. Calibration bar shows RhoA activation (Red) relative to basal RhoA activity (Blue). Graph on the right shows quantification of DORA RhoA biosensor ** $P < 0.01$ (ANOVA) (**a**) ** $P < 0.01$ control versus RhoA depletion (Student's t-test) (**b**). * $P < 0.05$ Saline versus IL-1β/TNFα (Student's t-test) (**e**). Data are representative of three independent experiments (**a-c**) 10 experiments (**f**) (error bars (**a-e**), s.e.m).

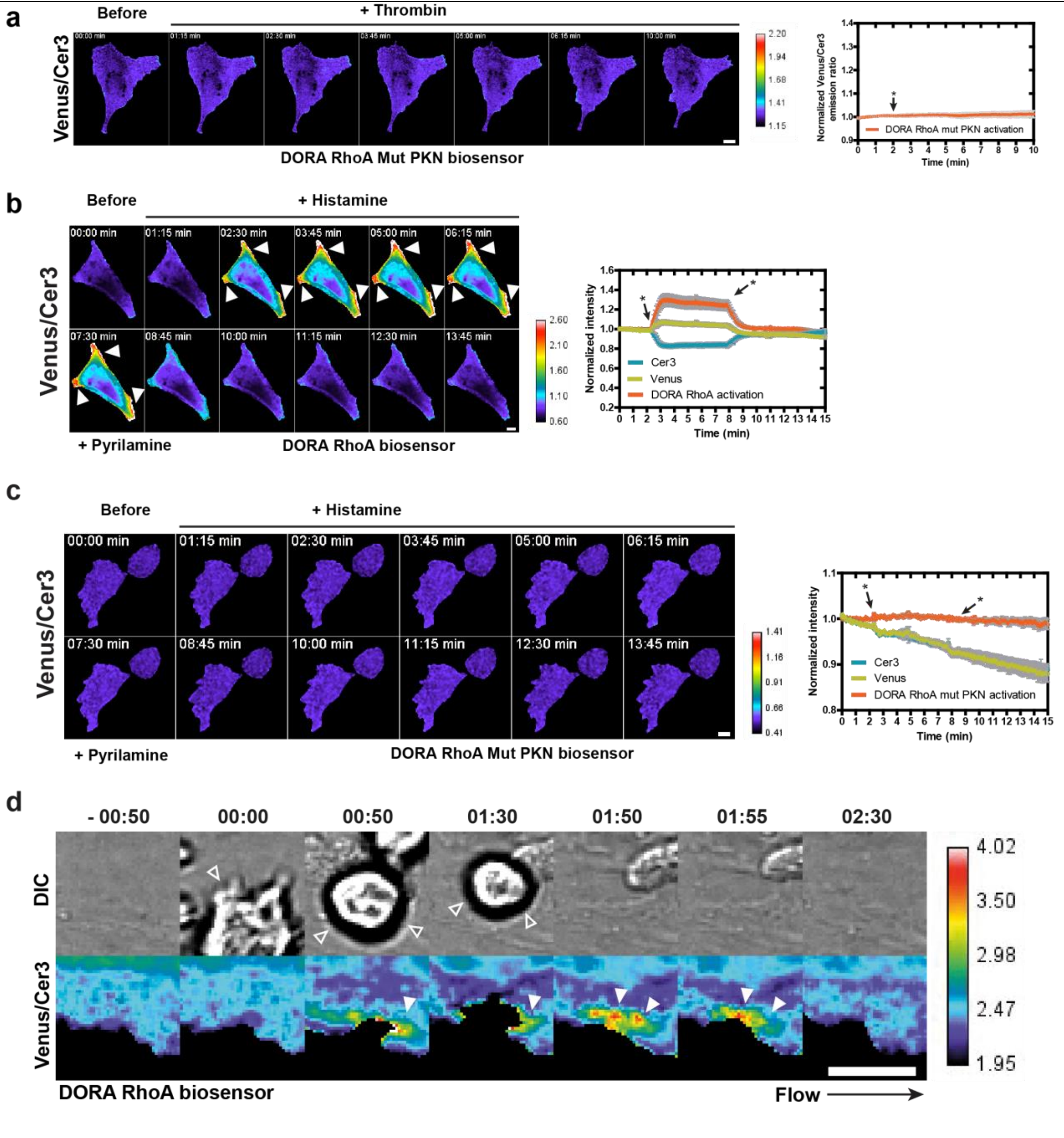

Supplementary Figure 2 Characterization of DORA RhoA biosensor and DORA RhoA mutant PKN biosensor. (**a**) Time-lapse Venus/Cer3 ratio images of DORA RhoA mutant PKN biosensor after thrombin treatment in HUVECs. Scale bar, 10µm. Calibration bar shows RhoA activation (Red) relative to basal RhoA activity (Blue). Graph on the right shows quantification of DORA RhoA mutant PKN biosensor after thrombin treatment (asterisk) in HUVECs. (**b**) Time-lapse Venus/Cer3 ratio images of DORA RhoA biosensor or DORA RhoA mutant PKN biosensor (**c**) after histamine and subsequent antagonist pyrilamine stimulation in Hela cells expressing the histamine receptor. Filled arrows indicate RhoA activation. Graph on the right shows quantification of DORA RhoA activation. Asterisk indicate addition of histamine; second asterisk shows addition of pyrilamine. (**d**) Epi-fluorescent live-cell imaging of HUVECs expressing the DORA RhoA biosensor during neutrophil adhesion under physiological flow conditions (0.8dyne per cm^2). Open arrows indicate adherent neutrophil at the apical side of the endothelium. Filled arrows indicate local RhoA activation during neutrophil diapedesis. Scale bar 10µm. Calibration bar shows RhoA activation (red) relative to basal RhoA activity (blue). Data are representative of 10 experiments (**a-c**).

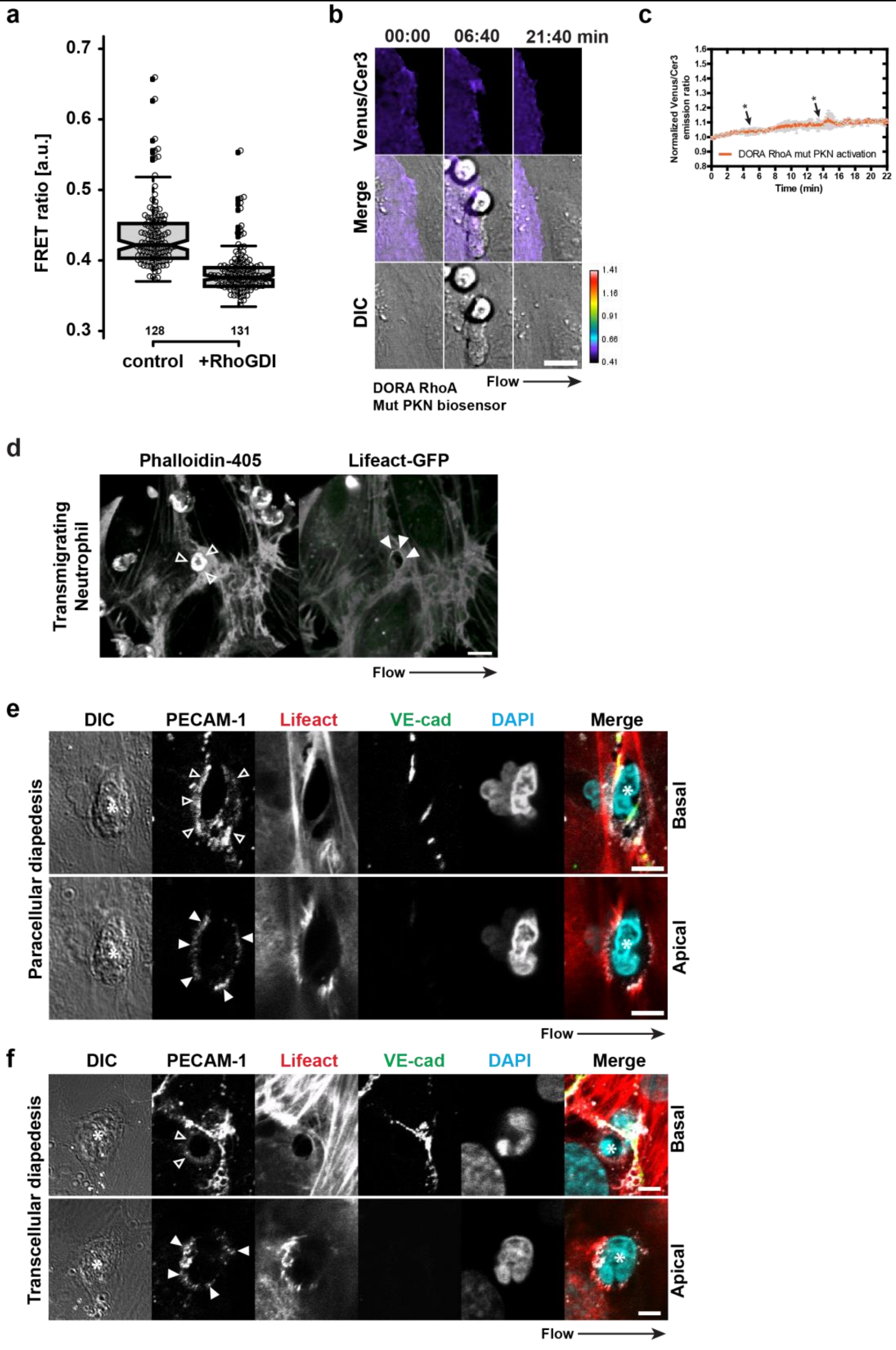

Supplementary Figure 3 F-actin rich endothelial pores. (**a**) Quantification of basal DORA RhoA biosensor ratios in Hela cells with and without RhoGDI overexpression. Box plot shows median with 95% confidence interval (notches) and the limits of the box indicate the 25th and 75th percentiles; whiskers extend 1.5 times the interquartile range from the 25th and 75th percentiles. The Venus/Cer3 ratios of individual cells are plotted as open circles. The lower FRET ratio in presence of RhoGDI reflectes reduced GTP-loading, which is in agreement with regulation by RhoGDIs. (**b**) Time-lapse Venus/Cer3 ratio images of DORA RhoA mutant PKN biosensor during neutrophil transmigration. Scale bar, 10µm (**c**) Quantification of DORA RhoA mutant PKN biosensor activation during neutrophil TEM. First asterisk indicates leukocyte adhesion; second asterisk shows leukocyte diapedesis. (**d**) Confocal imaging of extravasating neutrophils through Lifeact-GFP expressing HUVECs under physiological flow conditions, cells were fixed and stained for phalloidin. Open and filled arrows indicate F-actin visualized using phalloidin or Lifeact-GFP, respectively. Scale bar, 20µm. (**e**) PECAM-1 localization in para-and transcellular endothelial pores. Paracellular migration of monocytes. Open and filled arrows indicate PECAM-1 localization to the endothelial pore at the basolateral or apical site, respectively. Lifeact (red), VE-cadherin (green) and neutrophil nuclei (blue). Asterisk indicates transmigrating neutrophil in DIC. Merge shows all colours. Scale bar, 5µm. (**f**) Transcellular migration of monocytes. Open and filled arrows indicate PECAM-1 localization to the endothelial pore at the basolateral or apical site, respectively. Lifeact (red), VE-cadherin (green) and neutrophil nuclei (blue). Asterisk indicates transmigrating neutrophil in DIC. Merge shows all colours. Scale bar, 5µm. Data are representative of ten independent experiments (**a**) seven experiments (**c**) > 20 transmigration events (**e**, **f**) (error bars (**a,c**), s.e.m).

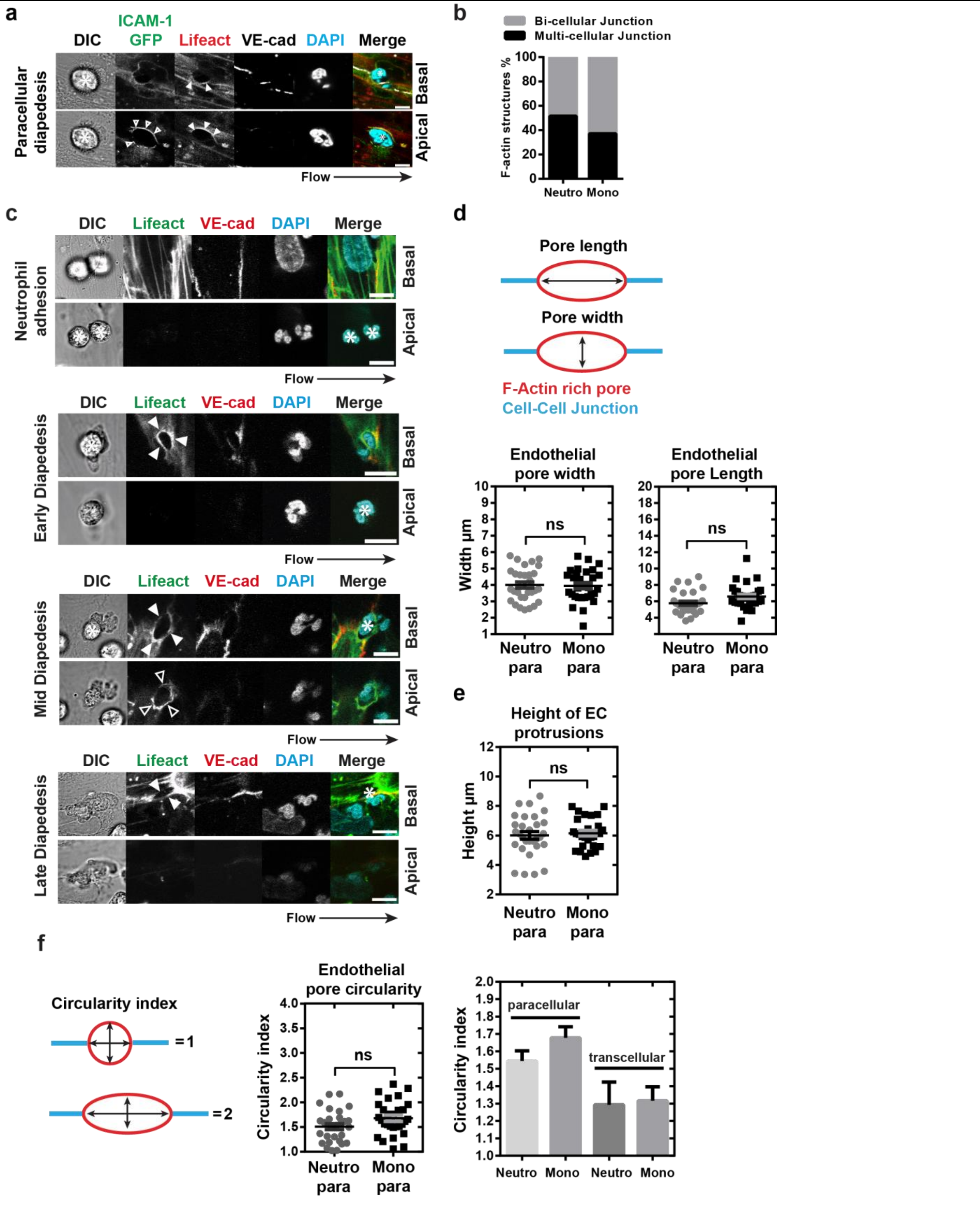

Supplementary Figure 4 Endothelial pore morphology at distinct stages of neutrophil diapedesis. (**a**) Confocal imaging of paracellular migrating neutrophils through ICAM-1-GFP and Lifeact-mCherry expressing HUVEC under physiological flow conditions. Open and filled arrows indicate ICAM-1-GFP and Lifeact-mCherry localization to endothelial pore, respectively. Asterisk indicates extravasating neutrophil (DAPI in blue). VE-cadherin (white). Scale bar, 5µm. (**b**) Quantification of paracellular migration route through bi-cellular (i.e. junctions of two adjacent ECs) or multi-cellular (i.e. junctions formed by three or more ECs). (**c**) Confocal imaging of Lifeact-GFP-positive endothelial pores at distinct stages of neutrophil diapedesis, defined as adhesion, early, mid, and late diapedesis. Open arrows indicate filopodia-like protrusions at the apical site of the structure. Filled arrows indicate the cortical actin-ring at the basolateral site that appeared during leukocyte crossing. Asterisk indicates extravasating leukocyte (DAPI in blue). Scale bar, 10µm. (**d**) Quantification of pore length and pore width defined as the distance parallel and perpendicular to the cell-cell junction, respectively. (**e**) Quantification of F-actin rich apical protrusions height for neutrophils and monocytes. (**f**) Quantification of endothelial circularity for para- and transcellular migrating neutrophils and monocytes according to the circularity index, Circle = 1 and oval = 2. Diagram on the left indicates the orientation of the pore for the paracellular route. Statistical significance was tested using a student's t-test. Data are representative of four independent experiments (**a**-**f**) with 40 transmigration events per group (error bars (**d**-**f**), s.e.m).

Supplementary Figure 5

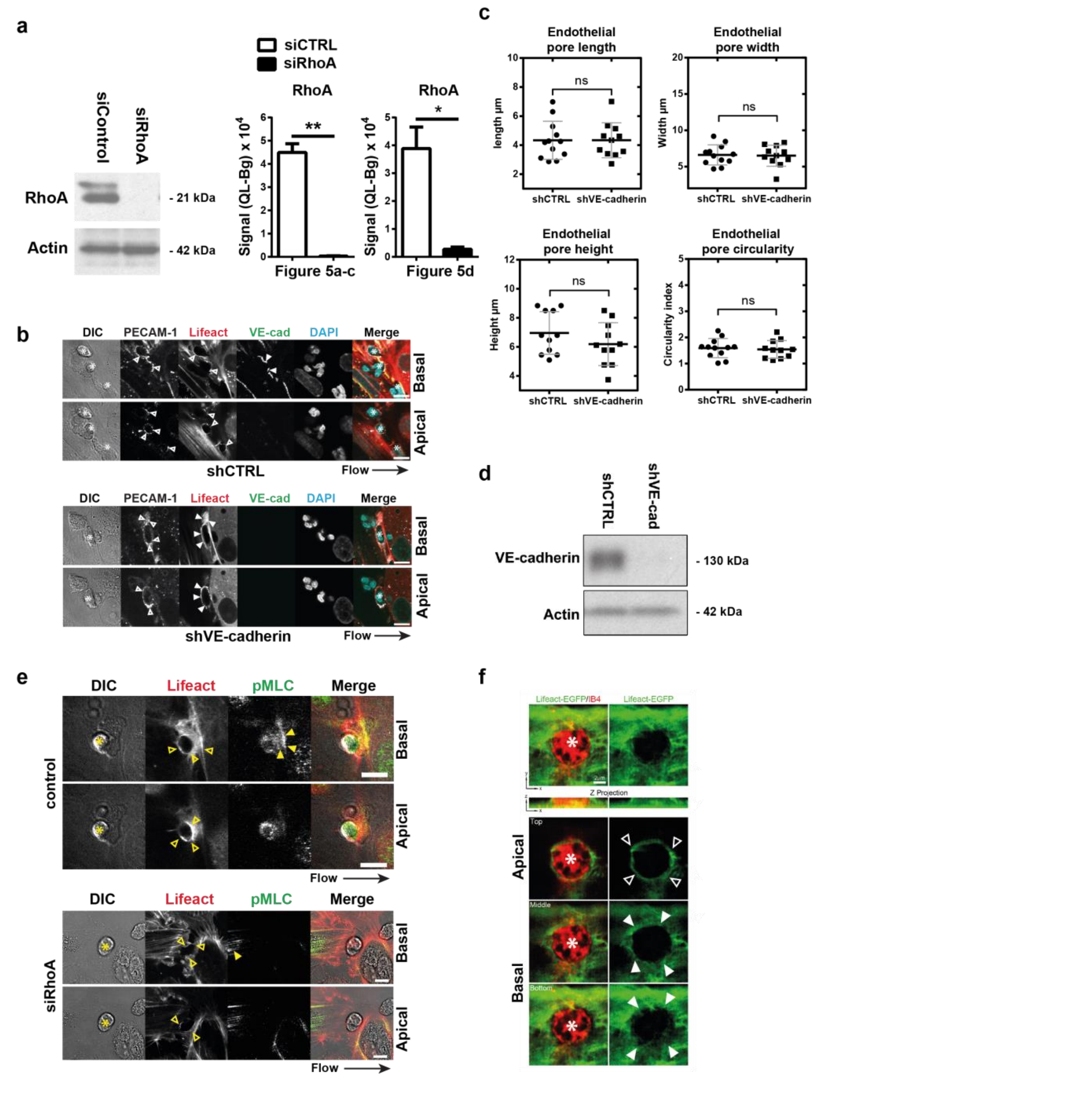

Supplementary Figure 5 RhoA signalling is required for local MLC phosphorylation and endothelial pore confinement during neutrophil transmigration. Endothelial RhoA and VE-cadherin depletion by siRNA. (**a**) Immunoblot analysis of protein extracts prepared from HUVEC treated with TNF-α overnight. Extracts were prepared 72 hours after transfection with control and RhoA siRNA duplexes. Extracts were probed with antibodies directed against RhoA and actin. Quantification of RhoA depletion, presented as ‘quantum level’ (sum of grayness of each pixel in blot) minus background (QL-Bg), normalized to that of actin. (**b**) Confocal imaging of paracellular migrating neutrophils through Lifeact-GFP expressing HUVEC after 72 hours transduction with control shRNA (left panel) or VE-cadherin shRNA (right panel) under physiological flow conditions. Open and filled arrows indicate PECAM-1 (white) and VE-cadherin (green) localization to the endothelial pore, respectively. Open and filled arrows indicate Lifeact-GFP localization to endothelial pore after 72 hours transduction with control shRNA (left panel) or VE-cadherin shRNA, respectively. Asterisks indicate extravasating neutrophils in DIC. VE-cadherin (green) and DAPI (blue). Merge shows all colours. Scale bar, 5µm. (**c**) Quantification of endothelial pore length, width, height, circularity of the endothelial pore in HUVECs after 72 hours transfection with control shRNA or VE-cadherin shRNA under physiological flow conditions. (**d**) Immunoblot analysis of protein extracts prepared from HUVECs treated with TNF-α overnight. Extracts were prepared 72 hours after transduction with control and VE-cadherin shRNA duplexes. Extracts were probed with antibodies directed against VE-cadherin and actin. (**e**) RhoA signalling is required for local MLC phosphorylation and endothelial pore confinement during neutrophil transmigration. Confocal imaging of paracellular migrating neutrophils through Lifeact-mCherry (red) expressing HUVECs after 72 hours transfection with control siRNA (upper panel) or RhoA siRNA (lower panel) under physiological flow conditions (0.8 dyne/cm^2). Open arrows in the apical and basal plane indicate filopodia-like protrusions at the apical site and the cortical F-actin-ring at the basolateral site of the endothelial pore, respectively. Filled arrows indicate MLC phosphorylation (green) localization during neutrophil transmigration under physiological flow conditions (0.8 dyne per cm^2). Asterisk indicates extravasating leukocyte in DIC. Scale bar, 10 µm. (**f**) Confocal imaging of F-actin dynamics during leukocyte diapedesis in retina vasculature of Lifeact-EGFP mice. Upper panel shows x-y and z-x projection of Lifeact-EGFP surrounding isolectin B4 (IB4) positive neutrophil. Open and filled arrows indicate Lifeact-EGFP localization to the endothelial pore at the basolateral or apical site, respectively. Asterisk indicates transmigrating neutrophil. Scale bar, 2µm. ** $P < 0.01$ and * $P < 0.05$ siCTRL vs siRhoA (Student’s t-test) (**a**).Data are representative of four independent experiments (**a-d**) with >12 transmigration events per group (**c**) (error bars (**a**), s.e.m, (**c**), SD).

Supplemental Figure 6.

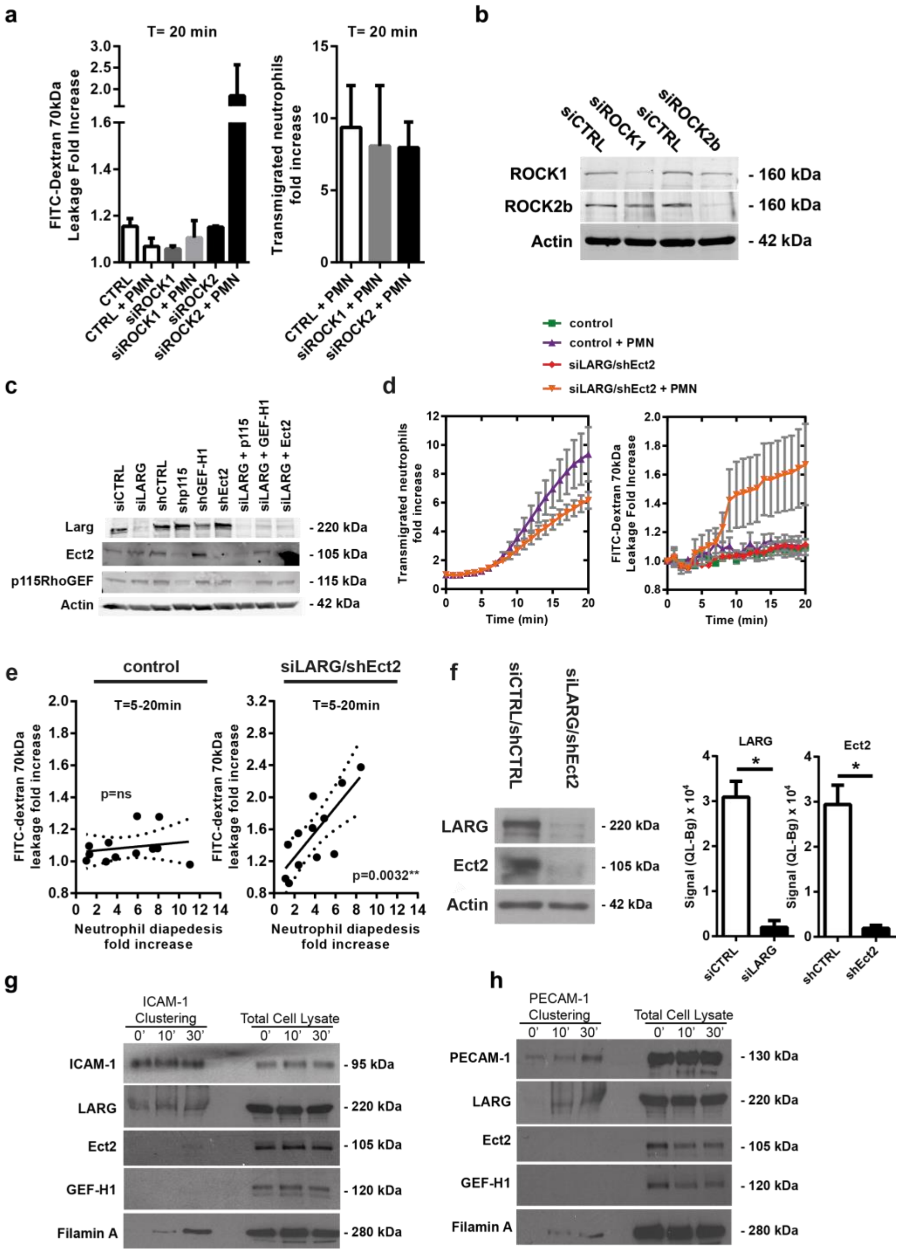

Supplementary Figure 6 LARG and Ect2 are recruited to intercellular tail of ICAM-1 upon clustering (**a**) Quantification of FITC-dextran and neutrophil extravasation after 20 minutes of neutrophil transmigration through control and ROCK1 or ROCK2b deficient ECs (**b**) Immunoblot analysis of protein extracts prepared from ECs treated with TNF-α overnight. Extracts were prepared 72 hours after transfection with control, ROCK1 or ROCK2b siRNA duplexes. Extracts were probed with antibodies directed against ROCK1, ROCK2b and actin. (**c**) Immunoblot analysis of protein extracts prepared from ECs treated with TNF-α overnight. Extracts were prepared 72 hours after transduction with control, Ect2, p115RhoGEF shRNA or transfection with LARG siRNA duplexes. Extracts were probed with antibodies directed against LARG, Ect2, p115RhoGEF and actin. (**d**) Extravasation kinetics of calcein-red labelled neutrophils and FITC-dextran through TNF-α treated ECs cultured on 3μm pore permeable filters. Neutrophils transmigrated towards a C5a chemotactic gradient in the lower compartment. Four conditions were tested; LARG/Ect2 deficient ECs + neutrophils (Orange line), control + neutrophils (purple line), LARG/Ect2 deficient ECs only (red line) and control only (green line). (**e**) Correlation analysis of dextran and neutrophil extravasation kinetics through control and LARG/Ect2 deficient ECs. (**f**) Endothelial LARG/Ect2 depletion by RNAi. Immunoblot analysis of protein extracts prepared from HUVEC treated with TNF-α overnight. Extracts were prepared 72 hours after transfection with control and Ect2 shRNA or transfection with control and LARG siRNA duplexes. Extracts were probed with antibodies directed against LARG, Ect2 and actin. Quantification of LARG/Ect2 depletion, presented as 'quantum level' (sum of grayness of each pixel in blot) minus background (QL-Bg), normalized to that of actin. (**g**) ICAM-1 clustering using beads coated with anti-ICAM-1 antibodies pre-and post-membrane lysis. Extracts were probed with antibodies directed against ICAM-1, LARG, Ect2, GEF-H1 and Filamin A. (**h**) PECAM-1 clustering using beads coated with anti-PECAM-1 antibodies pre-and post-membrane lysis. Extracts were probed with antibodies directed against PECAM-1, LARG, Ect2, GEF-H1 and Filamin A. $r = 0.1994$ $P = 0.5345$ (Pearson correlation) transmigrated neutrophils versus FITC-dextran leakage in control HUVECs or $r = 0.7734$ ** $P < 0.01$ (Pearson correlation) transmigrated neutrophils versus FITC-dextran leakage in LARG/Ect2 depleted ECs (**b**). * $P < 0.05$ siCTRL vs siLARG (Student's t-test) * $P < 0.05$ shCTRL vs shEct2 (Student's t-test) (**f**). Data are representative for three independent experiments (**d-h**) (error bars (**d-f**), s.e.m).

Supplemental Figure 7.

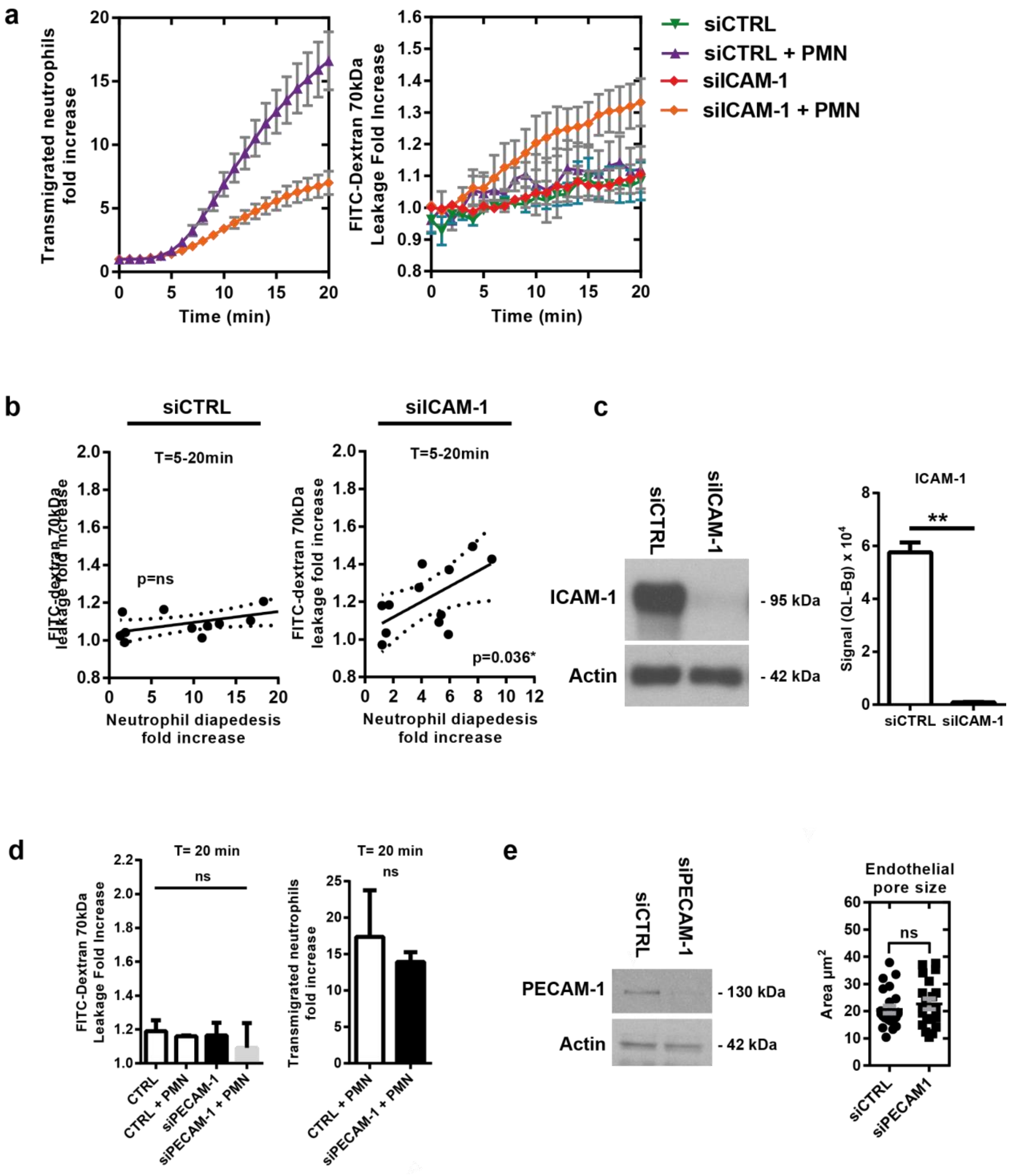

Supplementary Figure 7 ICAM-1, but not PECAM-1 depletion elicits vascular permeability during neutrophil diapedesis. (**a**) Extravasation kinetics of calcein-red labelled neutrophils and FITC-dextran through TNF-α treated ECs cultured on 3µm pore permeable filters. Neutrophils transmigrated towards a C5a chemotactic gradient in the lower compartment. Four conditions were tested; ICAM-1 depletion (EC) + neutrophils (Orange line), control + neutrophils (purple line), ICAM-1 depletion (EC) only (red line) and control only (green line). (**b**) Correlation analysis of dextran and neutrophil extravasation kinetics through control and ICAM-1 depleted ECs. (**c**) Endothelial ICAM-1 depletion by siRNA. Immunoblot analysis of protein extracts prepared from HUVEC treated with TNF-α overnight. Extracts were prepared 72 hours after transfection with control or ICAM-1 siRNA duplexes. Extracts were probed with antibodies directed against ICAM-1 and actin. Quantification of ICAM-1 depletion, presented as 'quantum level' (sum of grayness of each pixel in blot) minus background (QL-Bg), normalized to that of actin. (**d**) Quantification of FITC-dextran and neutrophil extravasation after 20 minutes of neutrophil transmigration through control and PECAM-1 deficient ECs. (**e**) Immunoblot analysis of protein extracts prepared from HUVECs treated with TNF-α overnight. Extracts were prepared 72 hours after transduction with control and PECAM-1 siRNA duplexes. Extracts were probed with antibodies directed against PECAM-1 and actin and quantification of endothelial pore size in control and PECAM-1 deficient ECs. $r = 0.5688$ $P = 0.053$ (Pearson correlation) transmigrated neutrophils versus FITC-dextran leakage in control HUVECs or $r = 0.6078$ * $P < 0.05$ (Pearson correlation) transmigrated neutrophils versus FITC-dextran leakage in ICAM-1 depleted ECs (**b**). ** $P < 0.01$ siCTRL vs siICAM-1 (Student's t-test) (**c**). Data are from three experiments (**a-d**) or are representative of > 20 transmigration events error bars (**a-e**) are s.e.m.

Supplemental Figure 8.

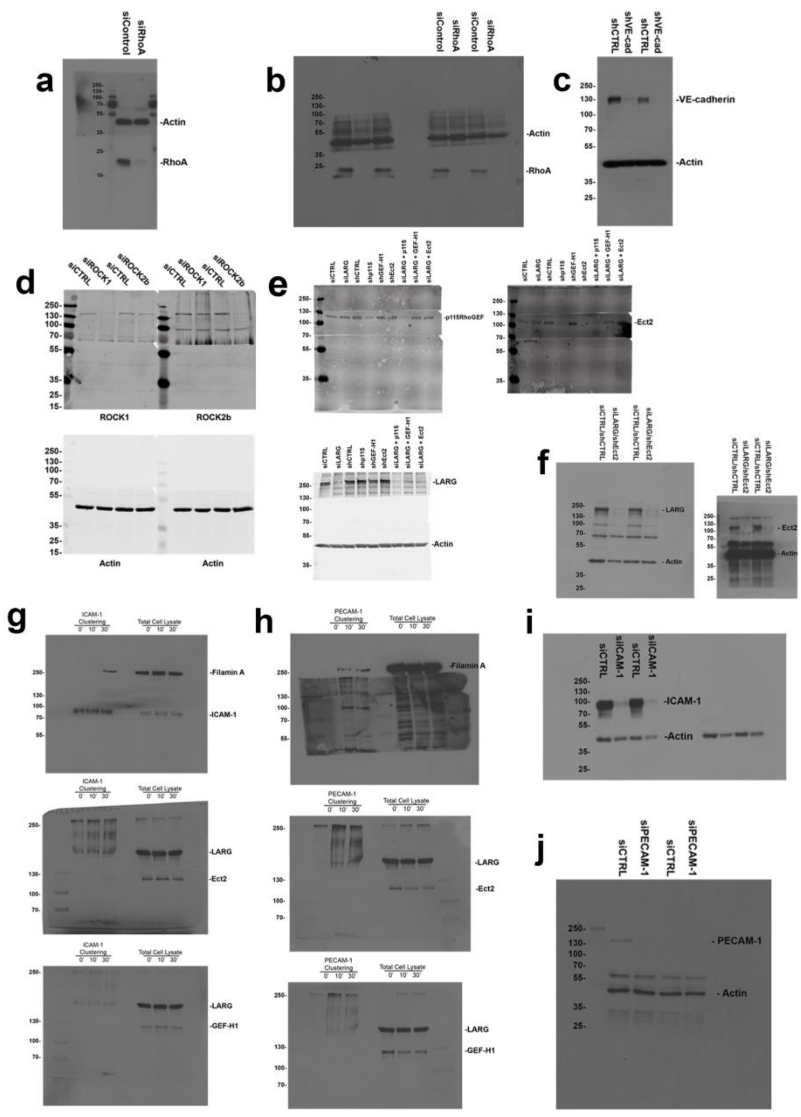

Supplementary figure 8. Full Western blots (**a**) Supplementary Figure 1b**:** Endothelial RhoA depletion by siRNA. Immunoblot analysis of protein extracts prepared from HUVEC treated with TNF-α overnight. Extracts were prepared 72 hours after transfection with control and RhoA siRNA duplexes. Extracts were probed with antibodies directed against RhoA and actin. (**b**) Supplementary Figure 5a: Immunoblot analysis of protein extracts prepared from HUVEC treated with TNF-α overnight. Extracts were prepared 72 hours after transfection with control and RhoA siRNA duplexes. Extracts were probed with antibodies directed against RhoA and actin. (**c**) Supplementary Figure 5d: Immunoblot analysis of protein extracts prepared from HUVECs treated with TNF-α overnight. Extracts were prepared 72 hours after transduction with control and VE-cadherin shRNA duplexes. Extracts were probed with antibodies directed against VE-cadherin and actin. (**d**) Supplementary Figure 6b Immunoblot analysis of protein extracts prepared from ECs treated with TNF-α overnight. Extracts were prepared 72 hours after transfection with control, ROCK1 or ROCK2b siRNA duplexes. Extracts were probed with antibodies directed against ROCK1, ROCK2b and actin. (**e**) Supplementary Figure 6c: Immunoblot analysis of protein extracts prepared from ECs treated with TNF-α overnight. Extracts were prepared 72 hours after transduction with control, Ect2, p115RhoGEF shRNA or transfection with LARG siRNA duplexes. Extracts were probed with antibodies directed against LARG, Ect2, p115RhoGEF and actin. (**f**) Supplementary Figure 6F: Endothelial LARG/Ect2 depletion by RNAi. Immunoblot analysis of protein extracts prepared from HUVEC treated with TNF-α overnight. Extracts were prepared 72 hours after transfection with control and Ect2 shRNA or transfection with control and LARG siRNA duplexes. Extracts were probed with antibodies directed against LARG, Ect2 and actin. (**g**) Supplementary Figure 6g: ICAM-1 clustering using beads coated with anti-ICAM-1 antibodies pre-and post-membrane lysis. Extracts were probed with antibodies directed against ICAM-1, LARG, Ect2, GEF-H1 and Filamin A. (**h**) Supplementary Figure 6h: PECAM-1 clustering using beads coated with anti-PECAM-1 antibodies pre- and post-membrane lysis. Extracts were probed with antibodies directed against PECAM-1, LARG, Ect2, GEF-H1 and Filamin A. (**i**) Supplementary Figure 7c: Endothelial ICAM-1 depletion by siRNA. Immunoblot analysis of protein extracts prepared from HUVEC treated with TNF-α overnight. Extracts were prepared 72 hours after transfection with control or ICAM-1 siRNA duplexes. Extracts were probed with antibodies directed against ICAM-1 and actin. (**j**) Supplementary Figure 7e: Immunoblot analysis of protein extracts prepared from HUVECs treated with TNF-α overnight. Extracts were prepared 72 hours after transduction with control and PECAM-1 siRNA duplexes. Extracts were probed with antibodies directed against PECAM-1.
